# Advances in sulfonyl exchange chemical biology: expanding druggable target space

**DOI:** 10.1039/d5sc02647d

**Published:** 2025-05-06

**Authors:** Lyn H. Jones

**Affiliations:** a Dana-Farber Cancer Institute Boston MA USA lyn_jones@dfci.harvard.edu; b Harvard Medical School Boston MA USA

## Abstract

Targeted covalent inhibitors possess advantages over reversible binding drugs, that include higher potency, enhanced selectivity and prolonged pharmacodynamic duration. The standard paradigm for covalent inhibitor discovery relies on the use of α,β-unsaturated carbonyl electrophiles to engage the nucleophilic cysteine thiol, but due to its rarity in binding sites, the amino acid is often not available for targeting. 10 years ago we highlighted the emerging potential of sulfonyl fluoride chemical probes that were initially found to serendipitously modify residues beyond cysteine, including tyrosine, lysine, histidine, serine and threonine. Since then, the rational application of sulfonyl fluorides and related sulfonyl exchange warheads to site-specifically target diverse amino acid residues in proteins using small molecules, oligonucleotides, peptides and proteins, has made considerable progress, which has significantly advanced covalent therapeutic discovery. Additionally, sulfonyl exchange chemistry has recently shown utility in the labeling of RNA and carbohydrates, further expanding the biomolecular diversity of addressable targets. This Perspective provides not only a timely update regarding this exciting area of research, thus serving as a useful resource to scientists working in the field, but areas of challenge and opportunity are highlighted that may stimulate new research at the chemistry–biology interface.

## Abstract 2

This Perspective assimilates developments in sulfonyl exchange chemical biology that have driven exciting advances in drug discovery and fundamental research over the last decade.

## Graphical abstract

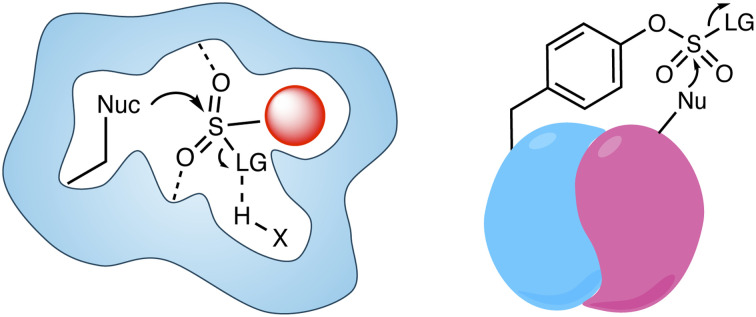

## Introduction

Many therapeutically relevant targets have been deemed ‘undruggable’, or at least ‘difficult-to-drug’ using small molecules for a variety of different reasons. Some may lack traditional deep pockets, while others may possess binding sites that are similar, or even identical, to other proteins, which hinders the development of potent and selective ligands. Moreover, there are many proteins that have been found to be ‘ligandable’ using structural bioinformatics or chemoproteomics-based methods, but this does not equate strictly to ‘druggable’ where the physicochemistry of the pocket may dictate challenges for the medicinal chemist to be able to balance the required pharmacokinetic–pharmacodynamic (PKPD) profile needed to deliver a drug with a sensible dose and therapeutic index. As a result, there has been a steady increase in the diversity of therapeutic modalities being explored in the drug discovery community with the objective of expanding the druggable proteome.

Targeted covalent inhibitors (TCIs) have become an important addition to the medicinal chemistry toolkit to help address challenging targets. TCIs possess a number of advantages over reversible binding ligands, the most obvious being the reaction between the small molecule and protein that drives a considerable increase in potency. It is not unusual for drug discovery programs to stall when reversible binding ligands developed for a target protein that possess high potency in biochemical or biophysical assays using isolated recombinant proteins, are only weakly active in cells. This drop-off in potency is often attributed to low unbound cellular levels of the drug, usually driven by physicochemical features that impart high non-specific binding to biomolecules, sequestration into lysosomes, or high desolvation energies that limit cellular permeation.^1^ Another reason might be due to high concentrations of potent endogenous binders such as metabolites that compete with the inhibitor for binding. Highly potent TCIs may overcome many of these issues through time-dependent increases in engagement of the target and thus enhanced temporal inhibition of the active site.

An electrophilic protein-reactive warhead is, by definition, a structural alert that may cause immune-related toxicities resulting from haptenization.^2^ However, context-specific latent electrophiles designed to label a residue only present in the target site may enhance safety by increasing selectivity against related off-targets.^3,4^ Additionally, a TCI may possess a prolonged PD duration if the target has a long half-life, because protein function only returns following its resynthesis.^5,6^ In these instances, intermittent dosing regimens of relatively high clearance TCIs are feasible, that may enhance therapeutic indices by reducing body exposure (area under the curve, AUC), although high free *C*_max_-driven toxicities such as hERG would still need to be understood and potentially managed.^7^

Finally, an overlooked advantage of covalent modalities is the ease with which occupancy biomarkers can be developed relative to reversible binding drugs.^3^ Covalent labelling of the protein aids its isolation and analysis, often using mass spectrometry (MS) based assays, that enables quantification of the amount of target occupancy by the drug. These assays may be performed not only in cell and animal models, but also in clinical ex vivo experiments, and they help us understand how much target engagement is needed to drive the desired functional pharmacological effect. Consequently, more precise PKPD predictions are possible that ultimately aid human dose projection and clinical translation of the drug candidate by ensuring that the mechanism under investigation is effectively tested in patients.^8,9^

**Fig. 1:**
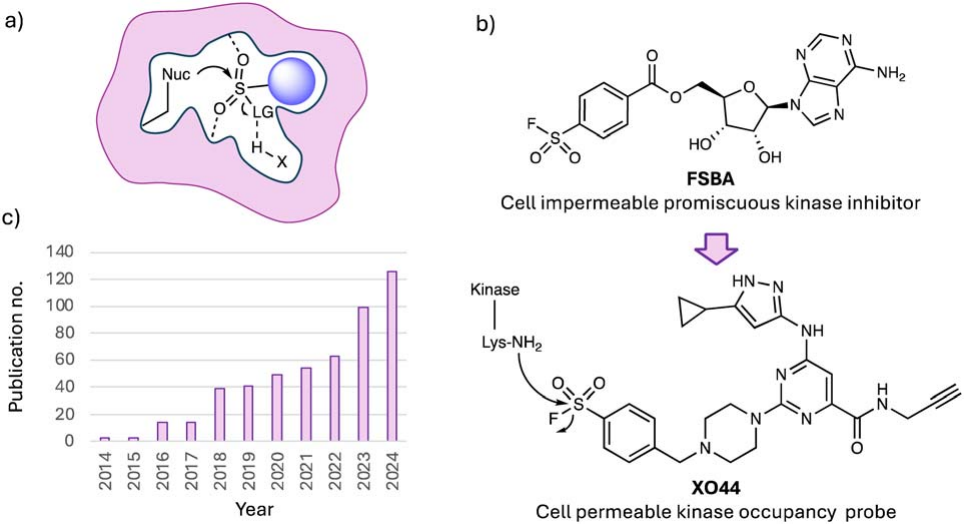
(a) Representation of a context-dependent site-specific reaction of a sulfonyl exchange warhead within a protein binding site illustrating interactions that enhance sulfur electrophilicity. (b) ‘SuFEx’ publication count (CAS Scifinder®). (c) Structures of sulfonyl fluoride chemical probes FSBA and XO44.

Despite these advantages, TCIs face two key limitations. The predominant paradigm relies on the use of acrylamide or butynamide electrophiles that target the nucleophilic cysteine thiol residue. Therefore, not only is the electrophilic warhead toolkit somewhat limited,^10^ but the scarcity of cysteine is a considerable drawback because it is usually not available for targeting in protein binding sites.^11^ About 12 years ago my lab realized this problem and started to explore ‘beyond cysteine’ targeted approaches that would enable a greater swath of protein binding sites to be addressed, and new modalities to be explored. We were particularly drawn to the pioneering studies of Bernard R. Baker, who was the first to put forth the concept of active-site-directed irreversible enzyme inhibitors,^12^ and who subsequently incorporated sulfonyl fluoride warheads into reversible binding molecules to convert them into irreversible ones.^13^ Sulfonyl fluorides had previously been determined to covalently target the catalytic serine in proteases by Fahrney and Gold,^14,15^ but it was Baker who harnessed this chemistry to successfully create a plethora of TCIs. The privileged reactivity of the warhead, preferring to react with a variety of nucleophilic amino acid side chains in the context of a binding pocket (Fig. 1a), whilst possessing quite surprisingly high aqueous stability, was then harnessed by Roberta Colman in elegant biochemical studies of nucleotide binding sites.^16^ Colman made many impactful contributions to covalent chemical biology, including the development of fluorosulfonyl benzoyl adenosine (FSBA, Fig. 1b), which was designed to map nucleophilic residues in adenosine binding pockets.^17^ FSBA was shown to covalently engage the conserved lysine in the ATP-site of kinases and thus became a useful promiscuous tool for studying the family, and served as inspiration for our development of the cell permeable probe XO44 (Fig. 1b),^18^ which will be described in detail later.

Baker and Colman, and many investigators since, have revealed the unique opportunities provided by sulfonyl fluoride medicinal chemistry and chemical biology, an area that we first reviewed in 2015 in this journal,^19^ but which has made considerable progress in the last 10 years. Our motivation to write the first Perspective was that we had recently exemplified our prototypical site-specific targeting of tyrosine residues using sulfonyl fluoride probes (details below),^20,21^ and we wanted to highlight the broader potential of this privileged electrophile. At a similar time, Sharpless and colleagues published a chemistry-focussed review that was also influential, approaching the area from the viewpoint of synthetic methodologies, describing sulfur–fluorine exchange (SuFEx) reactions as another example of click chemistry.^22^ Since then, there has been a steady increase in ‘SuFEx’ publications (Fig. 1c), including several reviews focussed on the synthetic utility of SuFEx chemistry.^10,22–24^

Importantly, new synthetic methods have also advanced the field by considerably enabling the preparation of a wide-variety of sulfonyl fluoride probes, which have traditionally been quite challenging due to the intrinsic reactivity of the electrophile.^23^ Early methods for incorporating sulfonyl fluorides into aromatic rings proceeded through fluorosulfonylation reactions that originally required the use fluorosulfonic acid, a highly corrosive reagent.^25^ More convenient methods were subsequently developed often employing simple chloride–fluoride exchange chemistry,^26^ including on-water biphasic syntheses using saturated aqueous solutions of KHF_2_.^22,27^ Since sulfonyl chlorides are themselves highly reactive and sometimes challenging to prepare, new methods employ oxidative chlorination–fluorination of thiols,^28–30^ or deoxychlorination–fluorination^31^ (or even direct deoxyfluorination)^32^ of sulfonic acids. More recently, advances in transition-metal catalysis have facilitated the sulfination of aryl bromides, iodides, and boronic acids, followed by electrophilic fluorination using Selectfluor, and related one-pot procedures.^33–36^

A recent review concisely highlighted the use of SuFEx chemistry to prepare compound libraries and the introduction of the warhead into biomolecules.^37^ I believe a timely Perspective is now needed to assimilate advances in chemical biology driven not only by SuFEx, but also other sulfonyl exchange chemistries from the last decade.

The Perspective is structured in a similar manner to our previous work,^19^ which groups examples from the literature by the specific amino acid side chains engaged by different chemical probes. The key difference is that in 2015 many of the described instances were fortuitous discoveries based on sulfonyl fluoride inhibitors that happened to modify a particular amino acid residue in a protein, but since then there has been a plethora of studies describing the rational, often structure-based design of covalent ligands that target sites in a wide variety of proteins. Most of the examples in this section describe the development of target-specific inhibitors and modulators, although reference is also made to kinase-directed activity-based proteomic probes, that have become quite well-established in the research community.

A new section has been added, namely Emerging Technologies and Therapeutic Modalities, which reflects the recent upsurge in novel approaches using sulfonyl exchange methodologies. This section emphasizes the growing impact of the area, particularly in drug discovery research, including breakthroughs in the development of covalent peptides, proteins and oligonucleotides. Throughout this Perspective I have aimed to provide a personal viewpoint of the field and how it has matured over the last 10 years since our first review of sulfonyl fluoride chemical biology.

## Tyrosine reactivity

**Fig. 2:**
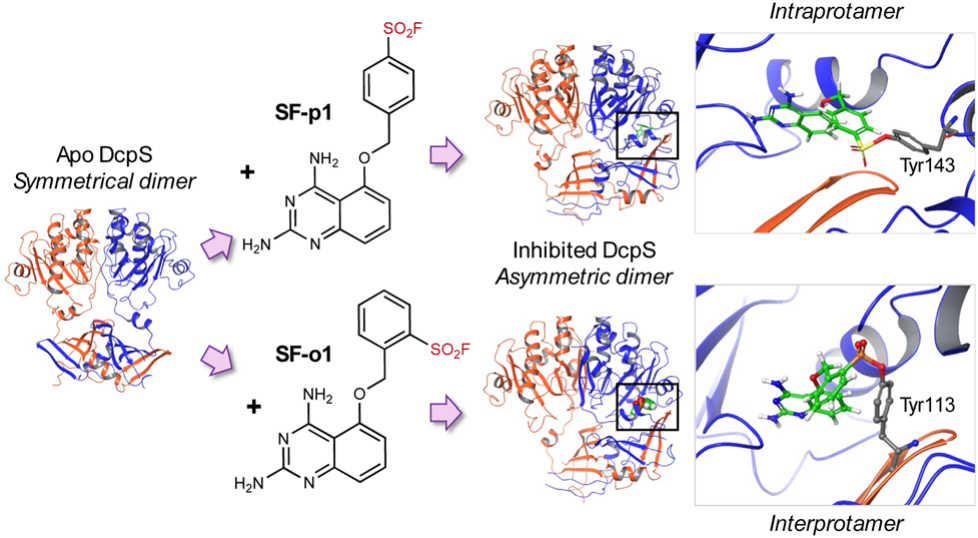
Intra- and interprotamer labelling of tyrosine residues at the surface of the catalytic pocket of DcpS by sulfonyl fluoride regioisomers SF-p1 and SF-o1 respectively. The DcpS homodimer is glued in an inactive asymmetric conformation by the inhibitors.

The first example of rational site-specific tyrosine targeting was published by our group in 2015. A sulfonyl fluoride electrophile was incorporated into a diaminoquinazoline inhibitor of the mRNA decapping scavenger enzyme DcpS, a target linked to spinal muscular atrophy and AML.^20^ The *para*-substituted derivative SF-p1 was designed to engage Tyr143 at the surface of the pocket within the homodimeric DcpS complex, glueing the enzyme closed in the inactive asymmetric conformation (Fig. 2). To demonstrate the context-dependent nature of residue modification using the SuFEx warhead, we moved the sulfonyl fluoride to the *ortho*-position (yielding SF-o1) to deliberately label Tyr113 at the surface of the partnering protomer.^20^ To my knowledge, this may be the first example of proximity-induced covalent modification in *trans* within a protein complex, effecting interprotomer targeting within the dimer. The sulfonyl fluorides were exceptionally potent (picomolar) inhibitors of DcpS, and an alkyne click handle was incorporated into SF-p1 to furnish a target engagement probe (SF-p1-yne) that enabled chemical biology validation studies in human primary cells.^38^ Our study also surveyed the microenvironment of labelled tyrosine residues found within the Protein Data Bank (PDB), which was later expanded upon in a more detailed structural analysis.^39^ Modified tyrosines appeared to be proximal to basic residues (lysine, histidine, and arginine) which likely enhance reactivity by facilitating deprotonation of the phenol. In the case of DcpS, Tyr113 and Tyr143 are proximal to His139 and Lys142 respectively, and additional hydrogen bonding interactions with the sulfonyl oxygens may also increase the electrophilicity on the sulfur atom.

This work served as the progenitor for a new drug discovery paradigm in our group that utilizes synthetic manipulation of protein surfaces to mediate induced-proximity pharmacology, exemplified using the E3 ubiquitin ligase adaptor cereblon described in the histidine targeting section below.^40^

**Fig. 3:**
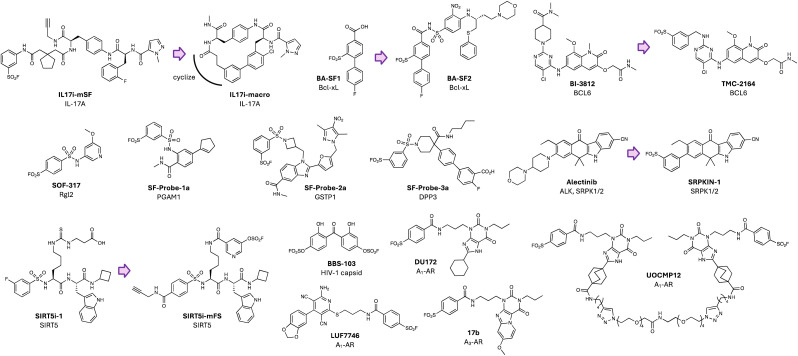
Tyrosine-targeting covalent chemical probes employing sulfonyl exchange warheads.

Following the success of our DcpS studies we started to apply the sulfonyl exchange platform to other projects within our portfolio. Among our published works during this time, the site-specific targeting of the proinflammatory interleukin-17A (IL-17A) is noteworthy from the perspective of advancing medicinal chemistry design strategies for inhibitors of protein–protein interactions, which are often challenging due to the shallow nature of the interfacial binding sites.^41^ In this case, the team had discovered a linear peptidic inhibitor of the interaction of IL-17A with its receptor IL-17RA,^42^ but in the early stages of the program we had an incomplete picture of the molecular mode-of-action of the ligand due to the lack of a crystal structure, which also hindered structure-based design. A computational model suggested the inhibitor might be bound in a site in IL-17A that appeared to be quite proximal to residue Tyr85. A clickable sulfonyl fluoride probe IL17i-mSF (Fig. 3) was designed to engage Tyr85, and its selective and complete modification was confirmed using intact mass and peptide mapping MS studies on the recombinant protein. This breakthrough validated a model in which the peptide would need to adopt a U-shaped conformation in the pocket and this insight led to the design of a macrocyclic reversible inhibitor IL17i-macro (Fig. 3) with improved potency and drug-like properties over the original linear peptide hit.^42^

It has been very pleasing to see these early applications of sulfonyl exchange chemical biology to drug discovery give way to advances in other labs that have successfully addressed challenging targets such as PPIs. For example, a sulfonyl fluoride warhead was incorporated into a biaryl fragment binder of the antiapoptotic protein B-cell lymphoma extra-large (Bcl-xL) to yield BA-SF1 (Fig. 3) that targeted Tyr101 of the BH3 loop (proximal to Arg100).^43^ Further optimization led to BA-SF2 (Fig. 3) that irreversibly inhibited Bcl-xL, with selectivity over related Bcl-2 that lacks the analogous tyrosine, and with improved cell-based activity over reversible inhibitors. In another example, site-specific covalent inhibition of the BCL6 oncogenic transcription factor was achieved through the incorporation of a sulfonyl fluoride into a reversible binder of the lateral groove pocket (BI-3812)^44^ that engages protein binding partners such as BCOR (Fig. 3). The chemical probe TMX-2164 (Fig. 3) was designed to modify Tyr58 (proximal to Arg28), which was confirmed by MS, and showed superior antiproliferative activity compared to reversible inhibitors.^45^ The enhanced cell-based potency of sulfonyl exchange TCIs is a general feature of irreversible inhibition, and the incorporation of such electrophiles could be applied more broadly to convert the ‘ligandable’ to ‘druggable’.

Ral (Ras-like) GTPases are directly activated by oncogenic Ras GTPases, and although cysteine-targeted covalent inhibitor drugs of mutant KRAS G12C have been developed, Ral and the majority of mutant RAS lack a ligandable cysteine. In a covalency-first approach, a screen of 89 sulfonyl fluoride-containing fragments identified a weak inhibitor of Ral (SOF-317, Fig. 3) which was anticipated and subsequently confirmed to engage Tyr82 which resides at the interface of Ral with the guanine exchange factor Rgl2, inhibiting this interaction.^46^ A crystal structure of the Ral-probe complex unexpectedly revealed a well-defined druggable cryptic site in the protein that had not been identified previously. This discovery formed the basis for optimization of more potent inhibitors with improved metabolic stability and activity in cancer cell invasion assays.^47^ These results demonstrate another potentially important feature of covalency – the likely ability to more readily identify and characterize cryptic sites that may enhance the chemical tractability of challenging targets lacking obvious deep pockets. Recently, DNA-encoded library (DEL) technology was applied to the creation of a 67-million-member library of sulfonyl fluoride containing small molecules.^48^ To illustrate the utility of the library, it was screened against three diverse enzymes, phosphoglycerate mutase 1 (PGAM1), glutathione S-transferase 1 (GSTP1) and dipeptidyl peptidase 3 (DPP3), and tyrosine-targeting inhibitors were identified (SF-Probe 1a, SF-Probe-2a and SF-Probe-3a respectively, Fig. 3). This approach has the potential to significantly advance covalency-first hit discovery screening strategies.

The development of selective kinase inhibitors is hindered due to the similarity of the ATP-site across the kinome family. To illustrate this, the ALK inhibitor drug alectinib (Fig. 3) was shown to inhibit several other kinases previously, including SRPK1/2 which regulate mRNA splicing and contribute to tumorigenesis.^49^ To develop a selective chemical probe of SRPK1/2 for target validation studies, a sulfonyl fluoride electrophile was rationally incorporated into alectinib to engage Tyr227 in the ATP-site. The irreversible inhibitor SRPKIN-1 (Fig. 3) was considerably more selective than alectinib, and only weakly inhibited ALK, as determined using competition chemoproteomics experiments.^49^ We have previously mapped the targetable ‘tyrosinome’ across the kinase family to illustrate opportunities for the development of additional selective TCIs using sulfonyl exchange chemistry.^50^

One of the main challenges of developing sulfonyl fluorides as chemical probes for *in vivo* applications, or as drug candidates, is their relatively high intrinsic electrophilicity that imparts stability issues, particularly in serum and plasma.^51^ The additional oxygen atom present within the fluorosulfate warhead attenuates this reactivity and significantly enhances metabolic stability, and the motif is thus finding utility in drug design strategies.^52^ Indeed, the stability of fluorosulfate has been used to develop ^18^F PET tracers for *in vivo* applications.^53–56^ In an example of rational TCI design, a fluorosulfate inhibitor of the lysine deacylase SIRT5 (a target linked to cardiac stress) was developed with considerably improved cell-based activity over its parent reversible binder (SIRT5i-1 *versus* SIRT5i-mFS, Fig. 3).^57^ The probe SIRT5i-mFS was designed to modify Tyr102, proximal to Arg105, within the substrate binding active site. This covalent peptidic inhibitor likely possessed inadequate bioavailability for oral dosing but showed requisite serum stability to achieve *in vivo* SIRT5 target engagement in the hearts of mice when delivered intravenously. Although the covalent inhibitor was rapidly cleared from blood, medicinal chemistry optimization (*cf.* macrocyclization of IL17i-mSF) may deliver more advanced inhibitors for pharmacological validation.

A recent covalency-first screen of a library of 472 fluorosulfates identified hit molecules that modified the HIV-1 capsid protein and inhibited its assembly.^58^ BBS-103 (Fig. 3) was found to serendipitously engage Tyr145 (proximal to His62 and Arg162) in the CAP-1 binding site and had antiviral activity in cell-based assays by perturbing virus production. These results suggest that larger libraries of weakly-reactive fluorosulfates with greater chemical diversity may find utility as a general approach for hit generation in drug discovery. A phage display peptide library is described below in the emerging technologies section that exemplifies such an opportunity.^59^

Other examples of fortuitous tyrosine modification have been reported using SuFEx chemical probes. For instance, the sulfonyl fluoride DU172 (Fig. 3) was developed previously as a potent irreversible antagonist of the A_1_-adenosine receptor (AR) GPCR.^60^ DU172 was found to significantly stabilize A_1_-AR to enable cryo-EM structural biology analysis of the protein, which showed that the warhead had modified Tyr271^7.36^.^61^ Further work delivered the sulfonyl fluoride 17b (Fig. 3) that irreversibly antagonized the related A_3_-AR through Tyr265 modification (analogous to the A_1_-AR Tyr271).^62^ Other covalent modalities incorporating sulfonyl fluoride electrophiles have been developed for the adenosine receptors, including the A_1_-AR partial agonist LUF7746 that persistently activates the receptor (*E*_max_ 60%) by targeting Tyr271,^63^ and bivalent ligands bearing two warheads that putatively bind across the A_1_-AR homodimer, with improved selectivity over A_3_-AR (UOCMP12, Fig. 3).^64^ A sulfonyl fluoride covalent antagonist of A_2A_AR was reported previously which was found to fortuitously label Lys153 (see following section for examples of rational design of lysine-reactive probes).^65^

The works described above illustrate the diverse nature of targetable tyrosine sites using sulfonyl exchange chemical biology across a variety of protein classes and binding sites. The emerging technologies section below further reveals the breadth of opportunity for covalent drug discovery through the site-specific targeting of tyrosine.

## Lysine reactivity

**Fig. 4:**
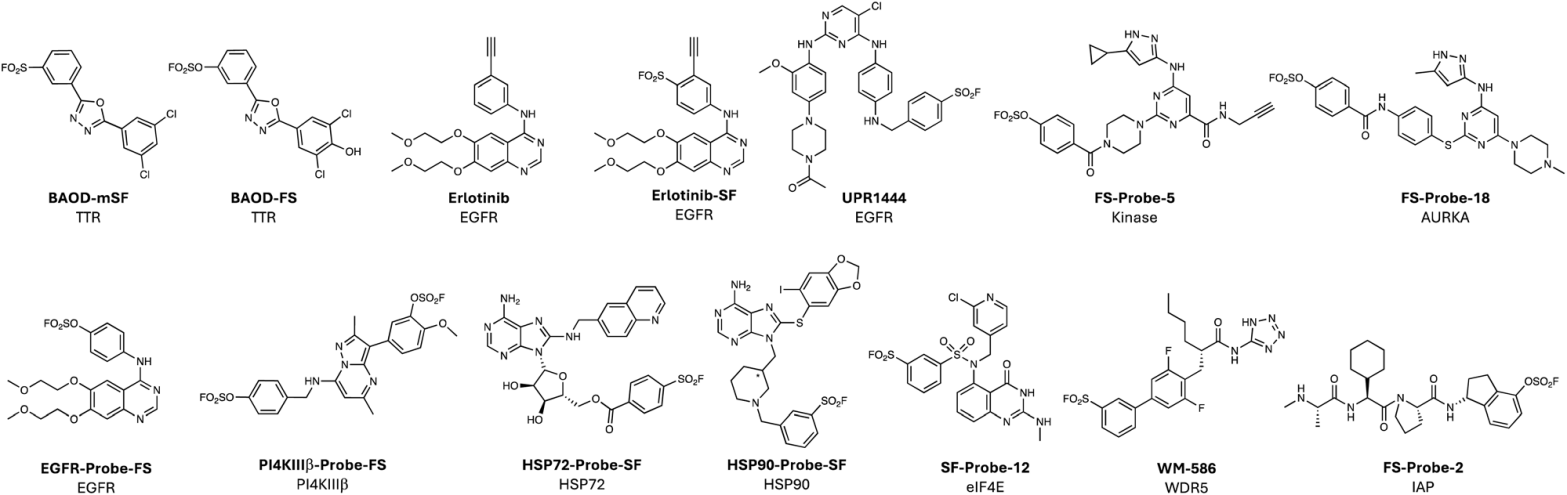
Lysine-targeting covalent chemical probes employing sulfonyl exchange warheads.

Sulfonyl exchange warheads are intrinsically oxophilic (hence their precedented reactions with tyrosine, serine and threonine), but the chemistry has also been very effectively applied to the rational development of lysine-targeting probes. In an early example, the sulfonyl fluoride BAOD-mSF, was designed to covalently modulate transthyretin (TTR) *via* modification of Lys15 in the thyroxine binding site.^66^ Interestingly, the latent fluorosulfate BAOD-FS (Fig. 4) also modified Lys15, though hydrolysis of the resulting adduct yielded the lysine-sulfate.^67^ Irreversible probes kinetically stabilized TTR and prevented the formation of amyloid fibrils that can cause polyneuropathy and cardiomyopathy. Lys15 is suggested to be p*K*_a_-perturbed because it is proximal to Lys15′ within the partnering TTR protomer, which would be expected to enhance nucleophilicity. Lys15 also forms a salt bridge with Glu54, and structural analysis suggested that the proton associated in this interaction may activate the electrophile by hydrogen bonding to the leaving fluoride ion. As expected, the microenvironment of lysine residues is an important consideration when attempting to target the side chain in protein binding sites.^39^ Intuitively, a significant depression of p*K*_a_ would be expected to enhance nucleophilicity by increasing the proportion of unprotonated amine, as seen for labelling of the N-terminus of sickle haemoglobin by the salicylaldehyde-containing drug voxelotor (in this case the p*K*_a_ is approximately 7.0).^68^ However, more research is clearly needed to fully elucidate the targetable lysinome,^50^ and chemoproteomics and computational methods may help these aims (see below).

Cysteine-targeting kinase inhibitors have shown considerable utility in drug discovery, but cysteine is not available for targeting in all ATP-sites, and in cancer the cysteine readily mutates to serine causing the covalent drug to lose efficacy, triggering relapse.^3,69^ The catalytic lysine is immutable and covalent drugs targeting this residue may address these issues, providing optimization of equilibrium binding interactions can provide the desired kinome selectivity.^70^ The catalytic lysine forms a salt bridge with a conserved glutamate in the kinase active state, and in the DFG-in conformation the conserved aspartate also forms a salt bridge with the lysine. Therefore, one might expect the targeting of the conserved lysine to be challenging due to the protonation of the side chain, although recent computational studies have suggested in certain conformations (DFG-out), and maybe through ligand binding, the p*K*_a_ of the residue is possibly reduced significantly.^71^ Nevertheless, inspired by the studies of Roberta Colman and co-workers that led to the development of FSBA as a promiscuous covalent kinase inhibitor (Fig. 1b),^16,72^ we reasoned that a more drug-like cell-permeable chemogenomic probe incorporating a minimalistic click enrichment handle would facilitate an assessment of kinase targetability mediated *via* covalent engagement of the conserved lysine in intact cells. The probe would also enable assessments of ATP-site occupancy, and thus selectivity, in living cells, as opposed to the previous use of biochemical panels employing recombinant proteins or methods using lysate, which do not accurately reflect the most physiologically relevant contexts of target engagement.^73^ In collaboration with the Taunton group, we developed XO44 (Fig. 1b), which incorporated into a promiscuous kinase inhibitor scaffold the sulfonyl fluoride warhead, to engage the catalytic lysine, and a terminal alkyne, that was strategically placed to avoid perturbing kinase binding.^18^ Subsequent computational modelling, informed by an EGFR/XO44 crystal structure, suggested that sulfonylation was further enhanced by hydrogen bonding from a sulfonyl oxygen atom to N–H backbone motifs in the P-loop.^74^ Cells treated with XO44 were then lysed, and protein adducts click conjugated to azido-biotin. Following streptavidin-mediated enrichment, trypsinization and MS proteomics, kinases labelled by the probe were inferred from the identified peptides. XO44 captured 133 kinases, a good proportion of those present in the Jurkat cell line used for the experiment, and competition proteomics experiments allowed for an assessment of in-cell kinase selectivity of dasatinib for the first time.^18^ XO44 has since been used widely in the community to determine the cell-based selectivity of several inhibitors, including PF-06873600 (a CDK2/4/6 inhibitor),^75^ a bitopic inhibitor DasatiLink-1,^76^ and NVP-BHG712 (an EphA2/4 inhibitor).^77^ Even though this technology was directed towards the assessment of kinase engagement, XO44 was able to enrich hundreds of non-kinases from cells, and unsurprisingly, the list was enriched for nucleotide-binding proteins. Additionally, a recent approach using XO44 in conjunction with phosphonate enrichment tags identified 715 liganded tyrosines in cells.^77^ These results suggest that chemogenomic probes such as XO44 may reveal targets that could provide new avenues for chemical probe and drug development (see emerging technologies section below).

XO44 also serves as a useful broad-spectrum tool to understand the changes in functional expression of kinases in cells. For example, lenvatinib is a multitargeted kinase inhibitor in clinical trials for hepatocellular carcinoma, and XO44 was used to understand potential resistance that may emerge to the candidate through kinase rewiring processes, as seen for other kinase inhibitor drugs.^78^ Through quantitative chemoproteomic profiling using XO44 it was revealed that CDK6 was significantly upregulated in lenvatinib-resistant HCC cells compared to mock controls, highlighting its role in the regulation of resistance. As a result, it was shown that CDK6 inhibition or degradation using PROTACs synergized with lenvatinib treatment.^78^

We also developed a TCI of the cancer target EGFR using erlotinib as the scaffold. A sulfonyl fluoride was incorporated into the inhibitor using structure-based design and the probe Erlotinib-SF (Fig. 4) was shown to selectively modify the conserved lysine.^79^ The terminal alkyne already present within erlotinib then served as a handle to perform click chemistry with azido-biotin that enabled the development of a target occupancy probe in living cells. A related covalent EGFR inhibitor bearing a sulfonyl fluoride warhead (UPR1444, Fig. 4) that engaged the conserved lysine was shown to possess superior antiproliferative effects against a clinically relevant triple mutant (L858R/T790M/C797S) over the acrylamide osimertinib that modifies C797.^80^

Others have followed the XO44 work and used alternative warheads to engage the conserved lysine. For example, a diverse set of sulfonyl fluoride and fluorosulfate versions of XO44, possessing a range of intrinsic reactivities, were used to explore and expand the targetable kinome (*e.g.* fluorosulfate FS-Probe-5, Fig. 4).^81,82^ These works confirm that an ameliorated electrophile can site-specifically label the conserved lysine. A non-clickable fluorosulfate FS-Probe-18 (Fig. 4) that labelled AURKA was shown to have a moderate oral bioavailability of 23% in rats, illustrating promise for the use of the warhead in drug development.^82^ Indeed, a fluorosulfate version of our sulfonyl fluoride erlotinib probe above also labelled the EGFR conserved lysine and possessed 32% oral bioavailability in rats (EGFR-Probe-FS, Fig. 4).^83^

A recent report described a further development of this strategy that incorporated two fluorosulfates into a reversible inhibitor of the PI4KIIIβ kinase that labelled the conserved lysine (Lys549) and a tyrosine (Tyr385) within the ATP-site, that was confirmed by X-ray crystallography (PI4KIIIβ-Probe-FS, Fig. 4).^84^ Interestingly, the probe appeared to lack activity in a biochemical assay, but a significant time-dependent increase in cell-based potency was observed, which was in-line with the slow rate of kinase labelling as shown by intact MS. Although further optimization of equilibrium interactions and enhancements to the rate of templated reactions with the warheads would be required to deliver a more effective inhibitor, these preliminary studies hold great promise for the development of highly selective and mutant-resilient TCIs, and the strategy could be applied to other protein targets.

There are several reported examples of rational lysine targeting beyond kinases, as might be expected from the unbiassed chemoproteomic profiling of XO44 described above. For example, several heat shock proteins were enriched by XO44, and coincidentally a TCI of HSP72 was developed subsequently by another group that engaged Lys57 near the ATP-binding site (HSP72-Probe-SF, Fig. 4).^85^ A surface exposed lysine on Hsp90 was targeted using the chemical probe HSP90-Probe-SF (Fig. 4) bearing a chiral, conformationally constrained linker that orients a sulfonyl fluoride warhead to react rapidly, and in an enantioselective manner, with the amine side chain.^86^ Interestingly, covalent modification of Lys58 Hsp90 promoted degradation of the protein, though *via* an unknown mechanism. Targeted protein degradation by TCIs has been observed in other studies and more work is needed to explore the underlying mechanisms at play.^87–89^

A rational covalency-first approach was described recently targeting eukaryotic translation initiation factor 4E (eIF4E), an mRNA cap-binding protein (like DcpS) that stimulates the translation of proteins involved in cancer cell proliferation and metastasis. The binding site is highly polar and previously reported reversible inhibitors are charged guanine derivatives with poor cellular permeability, which has hindered their development. An irreversible binder would be expected to circumvent these challenges, and although the cap binding site lacks targetable cysteines, Lys162 (which binds to the cap phosphate) appeared ligandable.^90^ The residue sits in a basic pocket and is flanked by lysine and arginine residues that likely further enhance nucleophilicity by reducing the Lys162 amine p*K*_a_.^91^ Covalent docking of more than 80k sulfonyl fluorides yielded 7 virtual hits that were acquired and screened for covalent binding to eIF4E. Structure-informed optimization of the most potent of two hits led to the identification of an inhibitor that was crystalized with the protein to enable further design. The chemical probe SF-Probe-12 (Fig. 4) possessed a higher rate of adduct formation with eIF4E and was the first inhibitor shown to possess cellular activity, albeit with weak potency, (likely compromised by the instability of the sulfonyl fluoride electrophile towards hydrolysis and glutathione-mediated reduction).^90^ Although this is clearly a proof-of-concept study, the work provides a starting point for the development of more efficacious TCIs with improved pharmacokinetics, and the methodology serves as a blueprint for hit discovery efforts using covalent docking and sulfonyl exchange chemical biology.

As described in the tyrosine targeting section above, sulfonyl exchange chemistry is well-suited to the development of PPI inhibitors. Interactions between the WBM site of WD repeat domain 5 (WDR5) and MYC are believed to play a role in cancer and inhibition of the PPI may have therapeutic potential. A high-throughput screen (HTS) of 330k compounds was performed at Novartis and reversible inhibitors of the WDR5-MYC interaction were discovered.^92^ The sulfonyl fluoride WM-586 (Fig. 4) was developed using SBDD that modified Lys250 in the WBM site, and inhibited the WDR5-MYC interactions, albeit weakly (IC_50_ 5.8 μM). More recently, an unbiased chemoproteomics-based methodology identified a probe for Tyr228 that also resides in the WBM site, which appears to be a more tractable starting point for optimization and is described below in the emerging technologies section.^93^

In another example of effective PPI inhibition, fluorosulfates targeting the inhibitor of apoptosis proteins (IAPs) were described recently that engaged a lysine within the BIR3 pocket of these proteins.^94^ The TCIs possessed cellular efficacy and prolonged plasma stability as expected, providing further evidence of the suitability of this warhead for drug development.

## Histidine reactivity

**Fig. 5:**
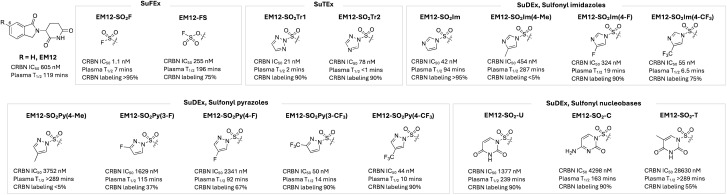
NanoBRET cell-based cereblon potency, plasma stability and CRBN labelling efficiency of rationally designed, covalent EM12-based chemical probes incorporating SuFEx, SuTEx and SuDEx warheads that site-specifically label His353 in the sensor loop of CRBN.

The first example of rational histidine targeting in a protein binding site using sulfonyl exchange chemical probes was reported recently by our group. Molecular glue degraders called immunomodulatory imide drugs (IMiDs), including thalidomide, lenalidomide, pomalidomide, and the isoindolinone congener of thalidomide called EM12 (Fig. 5), bind a pocket on cereblon (CRBN), an adaptor protein for an E3 ubiquitin ligase, and remodel its surface to induce proximity with neosubstrates, resulting in their targeted degradation. We reasoned that CRBN modulators that covalently engage residues at the surface of the pocket would mimic hotspot mutations and post-translational modifications (PTMs) that broadly drive the evolution of neoassociations in Nature, enforcing new surface physicochemistry that would induce the recruitment of non-canonical neosubstrates. However, the IMiD binding site does not contain a targetable cysteine residue, but a histidine (His353) at the tip of the so-called ‘sensor loop’ was within striking distance of the IMiD scaffold.^95^ EM12-SF (Fig. 5) was designed to engage His353 and very efficiently modified the residue in cells.^96^ Based on computational docking, the sulfonylated His353 was expected to inhibit recruitment of canonical neosubstrates that possess a distinct β-hairpin structural G-loop degron. Indeed, EM12-SF failed to degrade any protein significantly in cells (determined using expression level MS proteomics), and the probe has therefore found utility in target validation experiments by confirming the degradation mechanism-of-action of IMiD-based modulators through blocking of the CRBN binding site.^97^ Remarkably, switching to the fluorosulfate congener EM12-FS (Fig. 5), which also site-specifically modified His353, degraded a single protein, the N-terminal glutamine amidohydrolase NTAQ1, which is involved in the first step of the Arg/N-degron pathway.^96^ Interestingly, NTAQ1 had not been degraded previously by any reversible binding molecular glue or PROTAC, thus substantiating our original strategy of identifying novel neosubstrates using covalent neofunctionalization. Biochemical studies showed that degradation of NTAQ1 was reliant upon CRBN His353 covalent labelling, and we determined that the binding mode is novel since NTAQ1 does not contain a G-loop degron. Importantly, and as for many other neosubstrates, NTAQ1 was previously deemed undruggable, and so EM12-FS serves as the first pharmacological modulator that can be used to probe its biological function. This study exemplifies a potential new paradigm in drug discovery, that leverages neoprotein synthesis in cells to effect gain-of-function pharmacology using covalent small molecules.^40^ As mentioned above, sulfonyl fluorides and fluorosulfates are expected to possess different metabolic stabilities, and here we showed that EM12-FS was considerably more stable than EM12-SF in human plasma (Fig. 5) even though both molecules were stable in human liver microsomes and hepatocytes, which is due to the polarity of the molecules rather than specific metabolic vulnerabilities of the warheads.^98^

The sulfonyl triazole congeners EM12-SO_2_Tr1 and EM12-SO_2_Tr2 were shown to label His353 efficiently as expected, but they also suffered from poor plasma stability, similar to the sulfonyl fluoride (Fig. 5). Sulfur electrophilicity and leaving group ability were attenuated by developing a series of sulfur diazole exchange (SuDEx) warheads, and most derivatives retained very effective His353 modifying capability, and the sulfonyl imidazole in particular had very good plasma stability.^99^ These results show for the first time that not only fluorosulfates, but also sulfonyl imidazoles, and no doubt other SuDEx warheads, are suitable for drug development (Fig. 5).

A screen of covalent sulfonyl fluoride fragments prepared using a direct-to-biology methodology identified hits for BCL6 that engaged Tyr57,^100^ the same site of modification as the rationally designed inhibitor TMX-2164 described above.^45^ The screen also identified probes that engaged His115 in the same binding pocket, but at the opposite end to Tyr57.

The studies described in this section suggest that histidine should be considered alongside tyrosine and lysine as targetable residues using sulfonyl exchange chemistry. Indeed, histidine has a lower p*K*_a_ than lysine, and is usually deprotonated, which aids reaction with electrophiles.^101,102^ Additionally, histidine is frequently proximal to ligands in the PDB, and is the most prevalent nucleophilic catalytic residue in active sites, reflecting its intrinsic nucleophilicity in proteins.^103,104^ For these reasons, I believe we will see histidine-targeting increasing in popularity in covalent drug discovery research in the near future.

## Serine and threonine reactivity

**Fig. 6:**
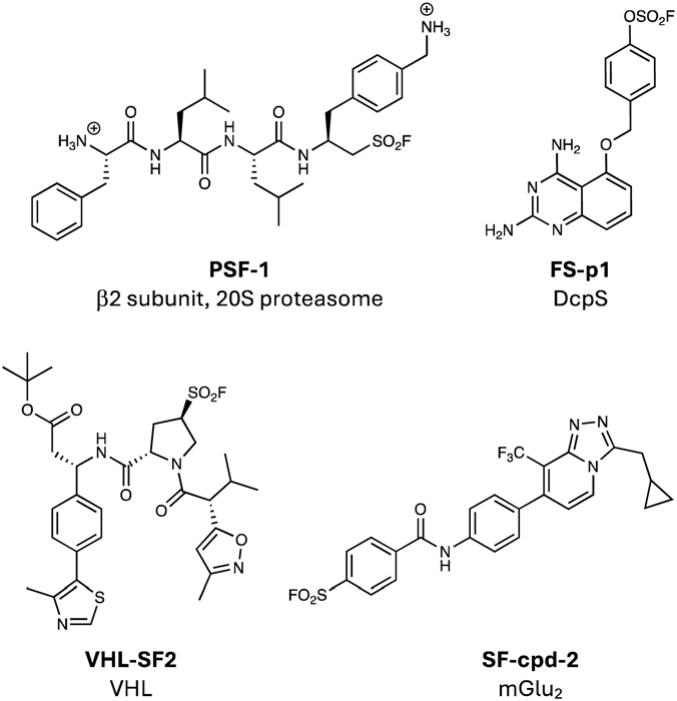
Serine targeting SuFEx probes PSF-1, FS-p1 and VHL-SF2, and threonine targeting SF-cpd-2.

The initial breakthrough in sulfonyl fluoride chemical biology arguably came in the 1960s when Fahrney and Gold discovered that chemical probes bearing the warhead covalently modified the catalytic serine in protease enzymes.^14,15^ Since then, many sulfonyl fluoride TCIs and chemical probes of proteases have been rationally developed, such as the selective covalent inhibitor of the β2 subunit of the 20S proteasome (PSF-1, Fig. 6).^19,105–108^ The nucleophilic serine resides within a catalytic triad with histidine and aspartate residues that enhance reactivity by 10^12^-fold. The microenvironment of the active site is clearly essential to the enhanced hydrolysis, which relies on histidine acting as a general base, although further insights into the complexity of the mechanism continue to be unearthed.^109^ The alcohol side chains of serine and threonine are intrinsically quite unreactive and therefore it is not surprising that there are very few reports of non-catalytic residues being targeted by electrophiles in proteins. We described the development of the fluorosulfate congener of SF-p1, *i.e.* FS-p1, that surprisingly engaged Ser272, a non-catalytic residue within the binding site of the DcpS enzyme.^110^ This was just the second report of non-catalytic serine targeting by a covalent inhibitor, the first report being the acetylation of Ser530 in cyclooxygenase by aspirin.^111^ Within the DcpS binding pocket, Ser272 is in proximity to a number of basic arginine, lysine and histidine residues that may perturb the p*K*_a_ of the nucleophilic hydroxyl group. We discovered during this work that the intact MS of the protein adduct was 18 mass units less than the parent, due to the elimination of the sulfonylated residue to the dehydroalanine species.^110^ We have observed this phenomenon for other serine and threonine labelled proteins and the presence of the eliminated dehydroamino acid in the MS can therefore be used as a useful diagnostic to report on the labelling of these residues.^110^ We previously suggested the potential utility of this chemistry to rationally create dehydroamino acids on proteins for uses in chemical and synthetic biology,^52,110,112^ and recently there have been reports of employing genetically encoded fluorosulfates for this purpose (also see emerging technology section).^113^ Additionally, FS-p1 was shown to possess improved metabolic stability over SF-p1 as expected, further highlighting the potential use of the warhead for drug development.^52^

Another recent report of site-specific serine targeting detailed the incorporation of a sulfonyl fluoride warhead into a VHL ligand to yield VHL-SF2 (Fig. 6) that was further developed into covalent PROTACs, which will be described in more detail below.^114^

At the time of writing this Perspective, there does not appear to be a published report of rationally targeting a non-catalytic threonine using sulfonyl exchange chemical probes. Previously, sulfonyl fluoride-containing positive allosteric modulators (PAMs) of mGlu_2_ were reported and one probe, SF-cpd-2 (Fig. 6), likely engaged Thr791 in the GPCR as suggested by computational modelling and receptor mutagenesis.^115^ The probe helped characterize the binding site for mGlu_2_ PAMs, which have potential utility for treating addiction. These results suggest that context-dependent labelling of threonine through rational design is likely feasible, as might the synthesis of dehydrobutyrine neoproteins (as for dehydroalanine).^112^

## Emerging technologies and therapeutic modalities

As the privileged nature of sulfonyl exchange chemistry has become better appreciated, there has been a considerable expansion in the breadth of its applications across chemical biology. Although the following section of the Perspective is not meant to be exhaustive, it serves to illustrate the significant growth and diversity of technologies and modalities that exploit sulfonyl exchange chemical biology for fundamental and translational research.

### Covalent anchoring using sulfonyl exchange (CASE)

**Fig. 7:**
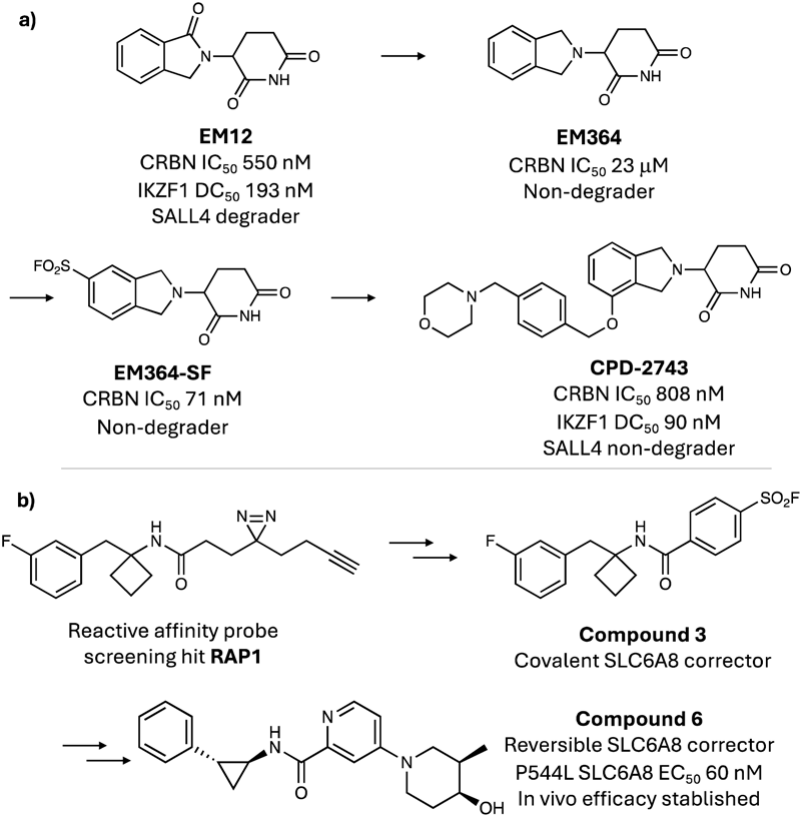
Covalent anchoring using sulfonyl exchange (CASE) strategy. (a) Scaffold hopping using sulfonyl fluoride reporters revealed a new series of isoindoline molecular glue degraders. (b) A screen of reactive affinity probes identified a ligand for the creatine transporter SLC6A8 (RAP1) that was converted to a covalent corrector of a trafficking mutant of the transporter (compound 3). Subsequent medicinal chemistry optimization yielded a potent reversible corrector (compound 6) that increased brain creatine levels in a mouse model of creatine transporter deficiency.

At the hit generation stage of drug discovery, weak-binding ligands are identified for a therapeutic target protein that serve as the starting points for medicinal chemistry optimization. Often at this early stage, small changes to the scaffold of the hit series will be detrimental to binding potency. This is particularly true for fragment hits because even small changes substantially affect binding, and such scaffold hopping approaches are frequently performed later in programs, which can limit a full exploration of the molecular design space. We reasoned that temporary electrophilic ‘reporters’, such as sulfonyl exchange warheads, incorporated into fragment hits would by-pass the need for optimization of equilibrium binding motifs and enable scaffold hopping at an earlier stage of hit-to-lead research. Once a new scaffold is identified, the covalent anchor would then be removed, delivering a new reversible binding series that would be distinct in its medicinal chemistry profile compared to the original hit series. We termed this approach covalent anchoring using sulfonyl exchange (CASE) and applied the method to CRBN as a case study. Through our successful exploits to synthetically modify the sensor loop His353 in CRBN, we hypothesized that the sulfonyl fluoride warhead could be incorporated into alternative weak-binding fragments to probe new structure–activity relationships.^116^ Subtle changes to the structure of the IMiD EM12 (including the removal of carbonyl groups, glutarimide alkylation or isoindolinone ring opening) obliterate CRBN binding, highlighting the importance of specific interactions within the thalidomide binding domain. When the sulfonyl fluoride warhead was incorporated into this set of molecules to label His353, only one rescued binding, EM364-SF, which possessed remarkably high CRBN potency in cells (Fig. 7a).^116^ This suggested that optimization of the equilibrium binding interactions of the parent isoindoline EM364 may deliver a new series of molecular glue degraders. Indeed, incorporating the benzyl morpholine tail from iberdomide, a highly potent IKZF1 degrader in clinical trials, into the EM364 scaffold yielded CPD-2743 (Fig. 7a), which retained IKZF1 activity and CRBN binding potency comparable to EM12. Importantly, CPD-2743 is differentiated from iberdomide in that it does not degrade SALL4, a neosubstrate linked to teratogenicity, and the molecule also possesses significantly improved permeability with no evidence of efflux, thus substantiating the effectiveness of the CASE strategy.

We recently reported a related study using sulfonyl fluoride probes to advance a series of correctors of trafficking mutations in the creatine transporter SLC6A8 that cause creatine transporter deficiency (CTD).^117^ A hit generation screen of a library of reactive affinity probes (RAPs) incorporating diazirine photo-crosslinking and click handle functionalities identified 40 specific binders of SLC6A8 in cells. The alkyne/diazirine motifs within these probes were replaced with sulfonyl fluoride reporters and one analogue, compound 3 (derived from RAP1), increased surface localization of the patient-derived P544L SLC6A8 variant 2-fold at 50 μM, providing confidence that the series may yield potent reversible binding correctors (Fig. 7b). Indeed, extensive medicinal chemistry optimization furnished ‘compound 6’ which increased cerebral creatine levels in a CTD mouse model. As for the CRBN pilot study described above, CASE was deployed here to effectively progress a new therapeutic modality for a challenging target class, helping to expand the druggable proteome.

### Mapping targetable sites using chemoproteomics

**Fig. 8:**
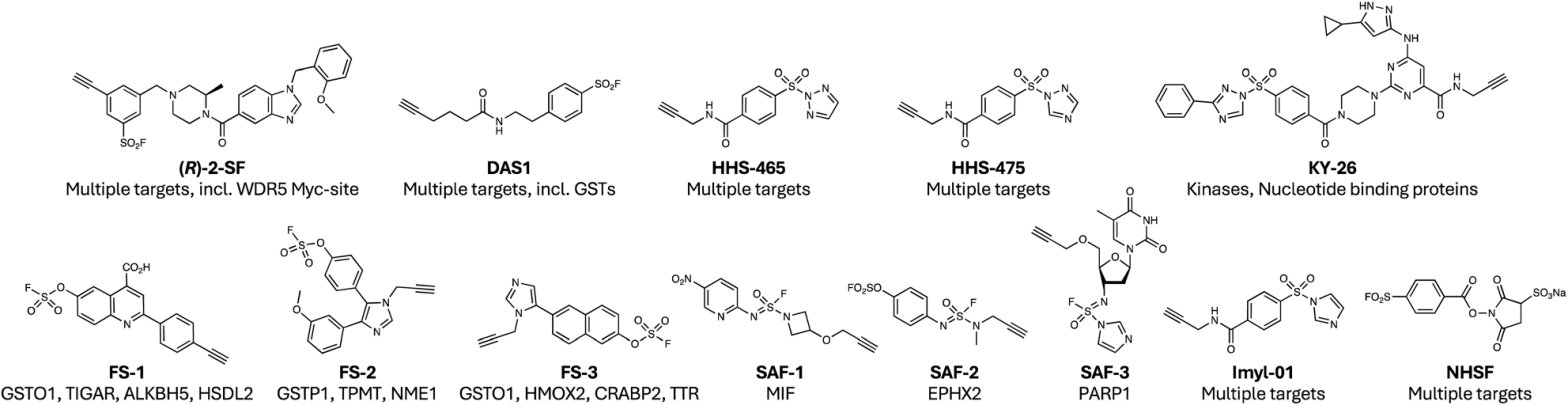
Chemoproteomic probes utilizing sulfonyl exchange chemistry.

Activity-based protein profiling, and related chemoproteomic methods using functional chemical tools, have significantly impacted drug discovery.^118,119^ Our studies described above using the semi-promiscuous lysine-targeting kinase probe XO44 (Fig. 1) nicely demonstrate the value of such methods.^18^ Motivated by these findings, and the ground-breaking work of the Colman group, we sought to further explore the binding proteins of privileged metabolite-based probes bearing sulfonyl fluoride and alkyne motifs using MS proteomics. Amongst the new discoveries provided by this study, we identified TCIs of nucleotide, amino acid and carboxylate transporters that have traditionally proven to be challenging drug targets.^120^ In contrast to these chemogenomic screening approaches, a more unbiased mapping of ligandable, and potentially druggable space was reported recently that employed sulfonyl fluorides incorporated into molecular scaffolds containing privileged drug-like motifs based on a methyl-substituted piperazine amide attached to heteroaryl diversity elements.^93^ Enantiomeric pairs of clickable covalent probes enabled stereoselective site-specific labelling of targets that were identified using MS proteomics. The enantioprobe technology provided high confidence ligandable hits, and probes were identified for several therapeutically relevant proteins, including (*R*)-2-SF (Fig. 8) that modified Tyr228 in the Myc-binding site of WDR5, inhibiting its pro-oncogenic protein interaction. In total, 513 ligandable tyrosines and 121 lysine sites were identified.

Even simpler probes have been developed that map hyperreactive sites in the proteome, placing less emphasis on equilibrium binding interactions, akin to the use of clickable iodoacetamide for cysteinome mapping. In an early example, DAS1 (Fig. 8) was shown to modify not only serine hydrolases as expected, but also functionally important tyrosines in GST enzymes.^121^ Several years later, similar minimalized probes (HHS-465 and HHS-475, Fig. 8) utilizing SuTEx chemistry were shown to enrich a wide swath of ligandable tyrosine and lysine sites from cells.^122,123^ The preference for SuTEx warheads to engage hyperreactive tyrosine residues translated to the suitability of the platform for the profiling of tyrosine phosphosites in the proteome.^122,124^ The features of the triazole leaving groups *versus* fluorine provides opportunities to further optimize equilibrium binding interactions with the target, and electronic properties may also be probed to modulate the intrinsic rate of reaction with proteins. The incorporation of simple recognition elements into the SuTEx probes has yielded numerous ligandable tyrosine sites across diverse proteins, including kinases (*e.g.* DGKα and ERK1/2), PTGR2, and G3BP1, all suitable for further medicinal chemistry optimization.^125–128^ A SuTEx analog of XO44, called KY-26 (Fig. 8), was also developed, that similarly mapped out several kinases and nucleotide binding proteins across the proteome.^129^ Although only ∼70 protein targets were identified using KY-26, compared to hundreds for XO44, technical optimization of the chromatographic and LC-MS/MS fragmentation conditions enabled identification of the probe-modified sites within these proteins, which included tyrosine residues in kinases, additional to the catalytic lysine.

Related chemoproteomic technologies have deployed electrophilic probes with attenuated reactivity compared to sulfonyl fluoride and triazole warheads, placing greater emphasis on context-dependent labelling through equilibrium binding and templated reactivity within the microenvironment of the binding site. Such an approach has been termed ‘inverse drug discovery’ by the Kelly group, referring to the use of more elaborate, potentially drug-like probes bearing latent reactive groups such as fluorosulfates to identify matched protein hits using chemoproteomics. Clickable fluorosulfates FS-1, FS-2, and FS-3 (Fig. 8) site-specifically engaged tyrosine and lysine residues across diverse proteins, each probe potentially serving as a starting point for lead optimization.

A similar approach was applied to a series of 16 sulfuramidimidoyl fluorides (SAFs, examples in Fig. 8), which are even weaker electrophiles than fluorosulfates.^130^ Of the 491 protein hits identified, 72% were distinct from those modified by sulfonyl fluoride and fluorosulfate probes previously, demonstrating the importance of the probe warhead chosen for target discovery using chemoproteomics.

Another latent reactive electrophile, the sulfonyl imidazole, that we had previously reported for site-specific targeting in CRBN (described above as an example of SuDEx^99^), was used for unbiased chemoproteomic profiling experiments in cells.^131^ A small set of clickable sulfonyl imidazole probes, including Imyl-01 (Fig. 8) identified 583 Tyr and 289 Lys sites across 439 proteins (>85% overlap with corresponding SuTEx probes). A SuDEx inhibitor of prostaglandin reductase 2 (PTGR2) was developed using SAR from a previous SuTEx effort and was found to have improved selectivity over GST (a common SuTEx and SuFEx off-target) compared to the corresponding triazole, suggesting that the warhead may be suitable for further development.^131^ Although metabolic stability testing was not performed, our previous study of CRBN SuDEx binders and this work validate the drug-compatible nature of the sulfonyl imidazole electrophile.^96,101^

A very simple heterobifunctional crosslinker employing an aryl sulfonyl fluoride and *N*-hydroxysulfosuccinimide (NHSF, Fig. 8) was developed for crosslinking mass spectrometry (CXMS) through a so-called ‘plant-and-cast’ strategy.^132^ The highly reactive NHS moiety first labels a protein surface lysine, and the sulfonyl fluoride warhead then labels proximal Tyr, Lys, His, Ser or Thr residues on neighbouring protein molecules. When applied to an *E. coli* lysate, 73 interlinked peptides were discovered. The low abundance of Lys–Lys crosslinks found in this study was presumably due to the relatively attenuated reactivity of sulfonyl fluoride compared to NHS with lysine. Conversely, the high frequency of Lys–Ser and Lys–Thr crosslinks reflect the success of the plant-and-cast method that accelerates through proximity the reaction with the weakly nucleophilic side chains. The development of a clickable probe may enable the capture and enrichment of a greater number of native protein interactions in whole proteomes. The latent reactivity of the fluorosulfate warhead has been applied to genetically encoded amino acids to crosslink proteins in cells, which is described below in the covalent protein section.

### Targeted protein degradation

**Fig. 9:**
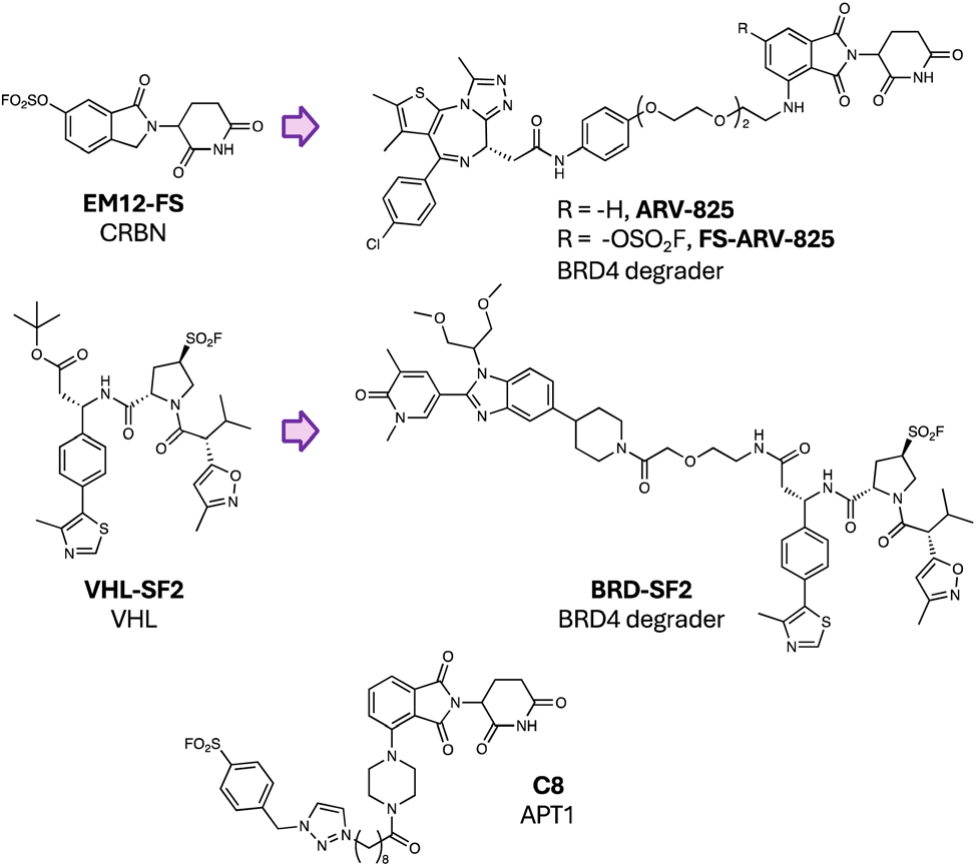
Covalent PROTACs using SuFEx warheads.

We reported the first design of a covalent E3 ligase binder through our work on His353 targeting the sensor loop of CRBN described above.^96^ The sulfonyl exchange chemistry we developed could clearly be applied to other E3 ligases to effect gain-of-function ubiquitination and degradation of novel neosubstrates using covalent molecular glues. We subsequently applied the approach to rationally design the first covalent PROTAC that labels the E3, FS-ARV-825, based on the reversible binding BRD4 degrader ARV-825, that linked pomalidomide to the bromodomain inhibitor JQ1 (Fig. 9).^133^ The fluorosulfate FS-ARV-825 degraded BRD4 in cells but was resistant to wash-out and competition with a potent CRBN ligand, unlike ARV-825. These data provide evidence that covalent CRBN PROTACs will possess prolonged pharmacodynamic duration *in vivo*. Since engagement of His353 prevents canonical neosubstrate degradation, FS-ARV-825 did not degrade molecular glue targets such as SALL4 (a zinc-finger transcription factor linked to teratogenicity), unlike ARV-825, demonstrating a further advantage of the modality. Additionally, only a small fraction of CRBN needs to be reprogrammed by the covalent PROTAC to mediate highly efficient catalytic degradation of the protein-of-interest (POI).^133^

Above we touched on a similar approach that recently utilized SuFEx chemistry to enable structure-guided replacement of the hydroxyproline motif in a VHL binder with a sulfonyl fluoride warhead.^114^ VHL-SF2 modified Ser110 in the E3 ubiquitin ligase, and the probe was elaborated into a covalent heterobifunctional PROTAC degrader of BRD4 (BRD-SF2, Fig. 9). These preliminary studies showed some signs of early success and have potential to help develop prolonged duration degraders. This work is a very rare example of serine targeting and serves to expand the chemical toolkit for TPD development. Effective PROTAC degraders that covalently engage the POI have also been described, even though in theory these molecules lack a catalytic mode-of-action.^134^ For example, a simple aryl sulfonyl fluoride warhead was linked to pomalidomide using a variety of chemical tethers that led to the discovery of C8 (Fig. 9), a potent degrader of the serine hydrolase APT1 that selectively modified the catalytic serine residue of the enzyme.^135^

**Fig. 10:**
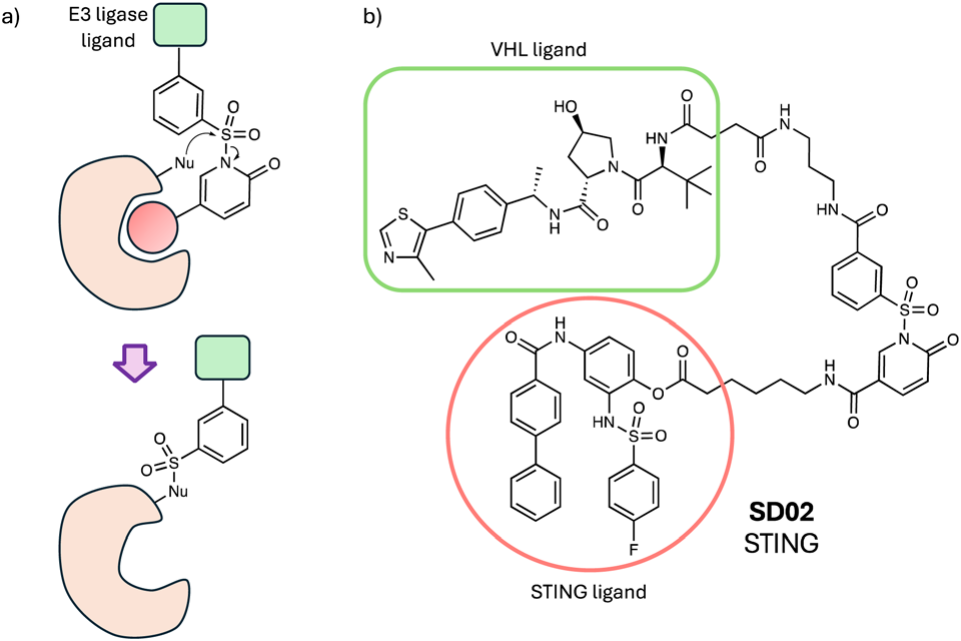
(a) Ligand-directed *N*-sulfonyl pyridone (LDSP) chemistry. (b) STING degrader utilizing LDSP.

Ligand-directed *N*-sulfonyl pyridone (LDSP) chemistry was developed previously to label protein surfaces using a sulfonyl exchange reaction (Fig. 10a).^136^ The proximity-driven chemistry enabled the labelling of two sites on the surface of carbonic anhydrase as a model system, Tyr7 (67%) and Lys169 (33%), that retained enzymatic activity, to create FRET-based biosensors *in situ*. More recently, the labelling technology was used to mediate the transfer of a VHL ligand to a tyrosine residue on the surface of the inflammation target STING, effecting its subsequent degradation.^137^ Although the degradation efficacy of SD02 (Fig. 10b) was somewhat modest, these preliminary studies appear to establish an alternative induced-proximity modality that may find broader utility in targeted protein degradation.^137^

In this section I have focussed on the opportunities for covalent small molecule-based degraders, while peptide and protein degraders using sulfonyl exchange chemistry are covered below.

### Covalent peptides and proteins

**Fig. 11:**
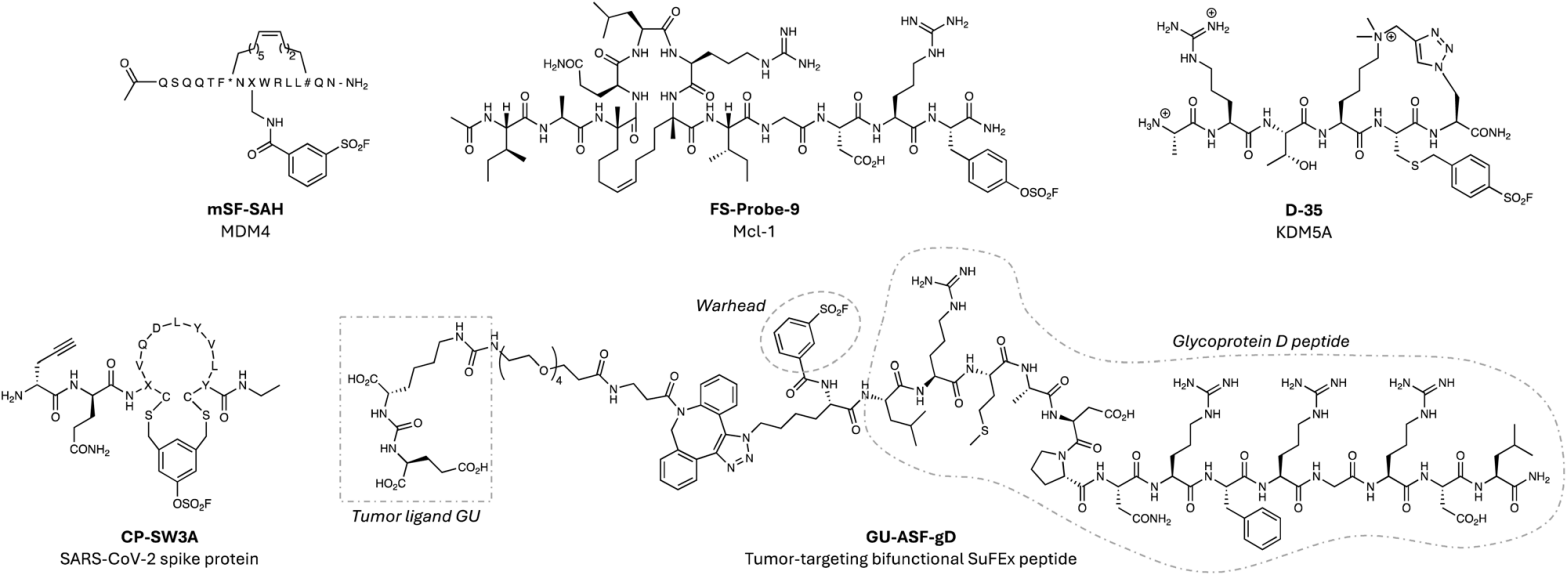
Covalent peptides using sulfonyl exchange chemistry.

Peptides are often starting points for the development of PPI inhibitors (such as IL-17A mentioned above) because protein interfaces are difficult to drug using small molecules. Increasingly, electrophilic warheads are now being incorporated into peptide modalities to enhance their pharmacodynamics, and sulfonyl exchange electrophiles enable targeting beyond cysteine (a residue often unavailable at such sites).^138^ In an early example of this strategy, the potency of a stapled peptide inhibitor of the interaction of MDM4 with the tumour suppressor p53 was improved 10-fold by incorporating an aryl sulfonyl fluoride motif through simple amide coupling (mSF-SAH, Fig. 11).^139^ Intact MS confirmed covalent engagement, potentially of proximal histidine or lysine side chains in the binding pocket, although this was not confirmed. Nevertheless, this pioneering work established the feasibility of readily converting peptidic disruptors of PPIs into more potent TCIs. A more recent example described the rational design of a fluorosulfate stapled peptide inhibitor of myeloid cell leukemia-1 (Mcl-1), a member of the BCL-2 family, which is a major resistance factor in cancer.^140^ Using structure-based design, a BH3-derived stapled peptide was armed with an aryl fluorosulfate to target His224 within the Mcl-1 binding site.^141^

The resulting potent inhibitor FS-Probe-9 (Fig. 11) was shown to rapidly engage His224 in a site-specific manner and possessed excellent buffer stability, although metabolic profiling was not reported. It will be interesting to see if the covalent peptide modality suffers from the cardiac toxicities that have plagued small molecule inhibitors of Mcl-1. The value of diverse fluorosulfate peptide libraries in hit discovery and covalency-first approaches are highlighted later in this section.

A sulfonyl fluoride electrophile was incorporated into a cyclic peptide binder of the PHD3 trimethyl lysine reader domain of the histone demethylase KDM5A, that engaged a surface-exposed lysine adjacent to the binding site (D-35, Fig. 11).^142^ Further work would be needed to convert this tool compound into a cell permeable inhibitor of the oncoprotein. The more attenuated reactivity of the fluorosulfate warhead was applied recently to the development of phage-display cyclic peptide libraries to enable hit discovery.^59^ To demonstrate applicability, the technology was used to identify an inhibitor of the interaction of SARS-CoV-2 spike protein with angiotensin II converting enzyme 2 (ACE2). The top covalent spike inhibitor CP_SW3A (Fig. 11) possessed irreversible antiviral activity, although the identification of the labelled site using MS was unsuccessful, reflecting a potential issue for the modality.^59^

SuFEx chemistry has also been used to stabilize macromolecular assemblies through the application of covalent synthetic peptides. An 18-subunit pore forming complex, CsgG:CsgF, has been used for DNA sequencing, and a sulfonyl fluoride warhead was incorporated into the CsgF peptide to enhance the stability of the 280 kDa complex.^143^ These so-called ‘SuTides’ react with CsgG monomers *via* proximity-enhanced crosslinking to a tyrosine side chain, creating homogeneous membrane channels suitable for future nanopore applications.

Covalent antibody recruiting molecules (cARMS) are synthetic bifunctional molecules that use a SuFEx electrophile to tether endogenous hapten-specific antibodies with tumour antigens on cancer cells.^144^ To show proof-of-concept, a native viral peptide epitope (from the herpes simplex virus gD domain) was armed with the sulfonyl fluoride or fluorosulfate warhead and a glutamate urea ligand for the prostate-specific membrane antigen (PSMA). The covalent peptides (*e.g.* GU-ASF-gD, Fig. 11) demonstrated enhanced anti-tumour immunotherapeutic activity compared to reversible binding peptide constructs.^144^ A related platform was developed recently that utilized affinity-induced covalent labelling of synthetic antigen receptors to enhance the activity of human T cells.^145^

**Fig. 12:**
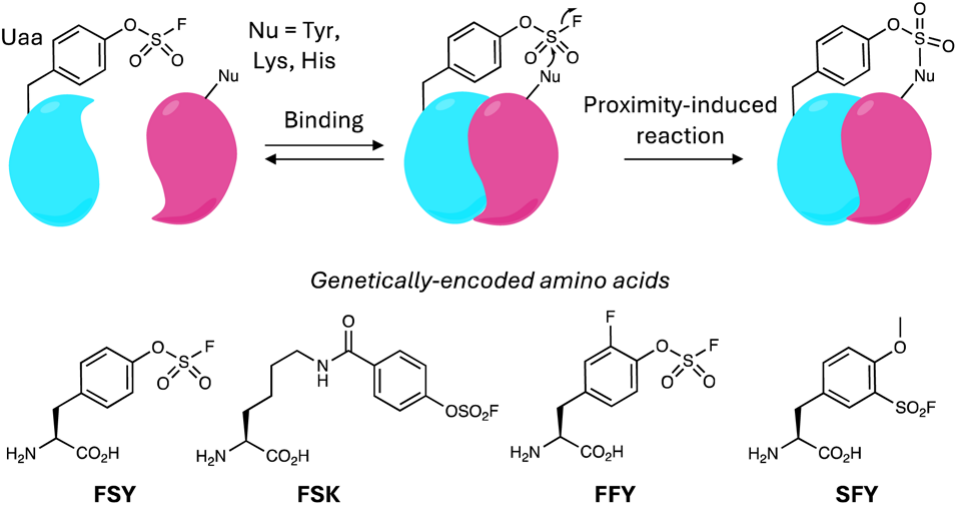
Covalent protein crosslinking using genetically-encoded unnatural amino acids (Uaa) incorporating SuFEx warheads.

As mentioned above, sulfonyl exchange chemistry has been utilized through genetic code expansion to facilitate protein crosslinks to explore protein complexes and enzyme substrates. A new tRNA-synthetase pair was evolved to genetically incorporate the fluorosulfate-l-tyrosine (FSY, Fig. 12) amino acid into specific sites on proteins.^146^ FSY mediated intra- and inter-protein crosslinks to proximal Tyr, Lys and His residues, capturing complexes between *E. coli* Afb and Z protein, as well as the complex between PAPS reductase and thioredoxin. To enable greater flexibility and longer reaction distance than FSY, another latent reactive unnatural amino acid, fluorosulfonyloxybenzoyl-l-lysine (FSK, Fig. 12), was genetically encoded into proteins in cells to enable crosslinking experiments.^147^ FSK and FSY were incorporated into thioredoxin, and MS proteomics was used to identify existing and potentially new substrates of the enzyme, and the different tags identified complimentary and distinct interacting proteins. The technology clearly demonstrates utility in mapping out protein complexes in live cells and serves as an orthogonal approach to other encoded chemically-induced crosslinking methods such as photoaffinity labelling.

Recently, the approach has been applied to the development of covalent protein therapeutics with enhanced pharmacodynamic profiles, so-called proximity-enabled reactive therapeutics (PERx, Fig. 12).^148^ Genetic incorporation of FSY into the ectodomain of PD-1 provided PD-1(129FSY) that covalently inhibited the endogenous PD-1/PD-L1 interaction by modifying His69 in PD-L1. PD-1(129FSY) enhanced T cell activation *in vitro* and inhibited tumour growth in mouse models with considerably higher efficacy compared to a non-covalent PD-1 protein, and similar efficacy to the antibody atezolizumab.^148^ The covalent protein is nearly an order of magnitude smaller than the monoclonal antibody, that may facilitate tissue penetration and accumulation, and has manufacturing advantages including high yield and low cost. In a similar manner, PERx was applied to the creation of a covalent nanobody that potently bound the SARS-CoV-2 spike protein and neutralized wild-type and several viral variants.^149^ The nanobody was engineered to contain an unnatural amino acid bearing a fluoro-substituted FSY (FFY, Fig. 12) that accelerated the reaction rate with the spike protein over FSY.

Another covalent nanobody technology was developed recently that was used to degrade cell surface proteins, exemplified using PD-1/PD-L1, as above. Firstly, FSY was incorporated into the anti-PD-L1 nanobody to target His69 on PD-L1 to create Nb-PD-L1-L108FSY, the so-called Gluebody.^150^ Subsequent conjugation of the Gluebody to a cell-penetrating peptide and lysosome-sorting sequence (CPP-LSS) yielded a GlueTAC that efficiently internalised and degraded PD-L1 *via* the endosome–lysosome pathway. The GlueTAC possessed superior T cell activation *in vitro*, and *in vivo* tumour growth inhibition over the Gluebody and atezolizumab, substantiating the enhanced efficacy of the covalent degrader. The approach complements other membrane protein degradation technologies, such as lysosome-targeting chimeras (LYTACs)^151^ and antibody-targeting chimeras (AbTACs),^152^ although the stability of the covalent attachment of the covalent nanobody to the surface antigen may avoid off-target effects during endocytosis.^153^

A covalent chimeric peptide-based targeted protein degradation platform (Pep-TACs) was recently reported that avoids the complexities of genetically encoding the unnatural amino acids.^154^ The Pep-TAC consists of a covalent peptide for the POI (PD-L1 in this case also) that was prepared using standard solid-phase peptide chemistry methods, conjugated to a peptide for the transferrin receptor TFRC that mediates delivery through lysosomal shuttling. The covalent peptide bearing a sulfonyl fluoride warhead increased residence time on PD-L1 (labelled residue not determined) to enhance degradation potency and pharmacodynamic duration. Surprisingly, the Pep-TAC was able to cross the blood–brain-barrier, albeit in mice, possibly due to the high expression of TFRC at the BBB, and the conjugate was able to inhibit brain tumour growth in a mouse model (superior to an anti-PD-L1 antibody).^154^ Further optimization of the Pep-TAC may include the use of more attenuated fluorosulfate or related electrophiles to enhance *in vivo* stability.

It is noticeable that several of the pioneering studies described above that utilize covalent therapeutic peptides and proteins leveraging privileged sulfonyl exchange chemistry, have used PD-L1 as a model system, and so it would be interesting to see side-by-side comparisons of their efficacy. Moving beyond the immune checkpoint case study will clearly be necessary to demonstrate the true breadth of opportunity afforded by these different platforms.

FSY has also been used as a reactive mimic of tyrosine-phosphate that served to elicit antibodies for the post-translationally modified residue, that have been traditionally challenging to develop. When Tyr612 of insulin receptor substrate 1 (IRS1) becomes phosphorylated it appears to mediate an interaction with p85α of PI3K that is involved in triggering downstream signalling, though high quality reagents that detect the modification are needed to further probe insulin stimulation pathways. IRS1 612-FSY was created using genetic code expansion and the new protein was then used to immunize mice.^155^ Antibodies were isolated and mAbs generated that crosslinked to the IRS1 612-FSY and were found to bind IRS1 phospho-Tyr612 and block its interaction with p85α.

Cancer targeted radioligand therapies have received considerable attention lately due to the approval of several therapeutics that effectively localize irradiation to the tumour. Improved efficacy may be achieved through prolonged tumour residence times, whilst a high blood clearance is necessary to maintain the desired therapeutic index. SuFEx chemistry was described in two recent reports as a means to deliver covalent radioligand therapies that anchor the therapeutic to the tumour site. Based on a structure of a nanobody-HER2 complex, FSY was encoded into position 54 of the nanobody in order to rationally target Lys150 in HER2, which is overexpressed on cancer cells.^156^ The covalent nanobody was conjugated to the α-emitter actinium-225 and was shown to be a potent inhibitor of tumour growth in a xenograft mouse model, while the wild-type noncovalent conjugated nanobody lacked efficacy.^156^ A related small molecule approach, that avoids the need for protein engineering, used FAPI-04, a DOTA-linked inhibitor of fibroblast activation protein (FAP) which is overexpressed on cancer cells. An aryl fluorosulfate warhead was attached to ^225^Ac-FAPI-04 to covalently engage tyrosine residues in FAP, and the radiopharmaceutical showed marked tumour suppression in a PDX model.^157^ The approach was also applied effectively to PSMA-targeted conjugates. It will be important to assess these covalent radiopharmaceuticals in detailed *in vivo* toxicology studies to see if these covalent strategies deliver enhanced therapeutic indices per their original objective.

### Oligonucleotide and carbohydrate-based technologies

**Fig. 13:**
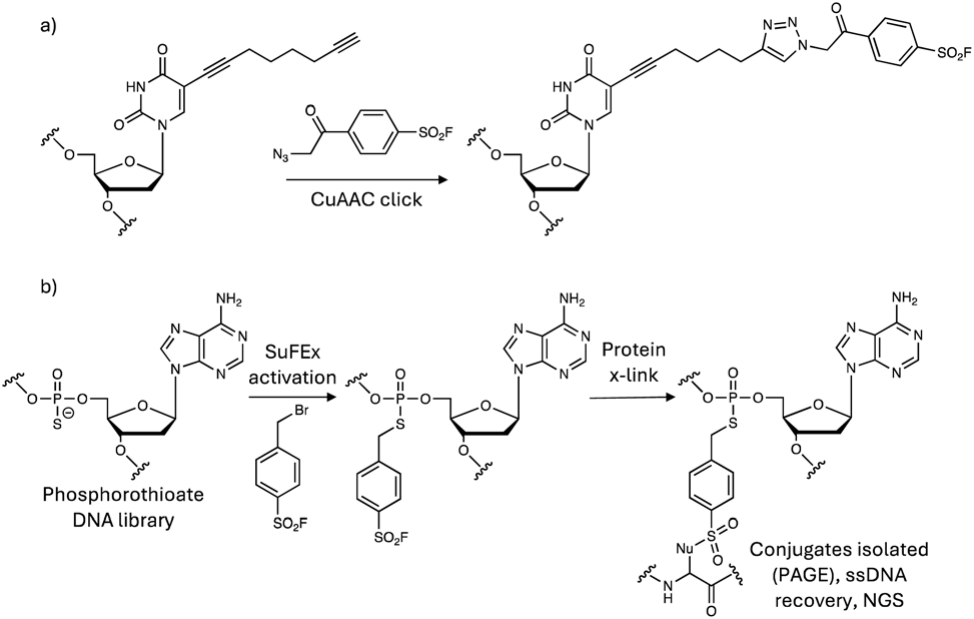
Synthesis of SuFEx covalent DNA aptamers using (a) click chemistry and (b) phosphorothioate alkylation strategies.

Sulfonyl exchange warheads can be incorporated not only into small molecules, peptides and proteins, but also oligonucleotides to enable site-specific crosslinking to binding proteins. An aptamer for thrombin was armed with a sulfonyl fluoride tethered to the oligonucleotide in a manner that would not clash with the thrombin interaction surface, using a click chemistry procedure (Fig. 13a).^158^ The covalent aptamer potently inhibited thrombin activity, but technical challenges prevented the use of MS analysis to identify the labelled residue within the protein–oligonucleotide complex. Instead, the disappearance of a specific peak in the trypsinized thrombin fragments mapped to a sequence containing tyrosine and histidine (IYIHPR) suggesting either of these may be the modified residues. More recently, sulfonyl fluoride-containing aptamers were conjugated to an LC3 ligand to mediate proximity-induced degradation of membrane proteins through autophagy/lysosomal clearance.^159^ The chimeric molecules provide another example of a platform targeted protein degradation technology enhanced through the use of sulfonyl exchange chemistry.

In a covalency-first approach, a sulfonyl fluoride-phosphorothioate nucleotide was designed to enable development of SuFEx *in vitro* selection allowing for the discovery of covalent protein inhibitors from trillions of SuFEx-modified DNA aptamers (Fig. 13b).^160^ The method identified disruptors of the interaction of SARS-CoV-2 spike protein with ACE2 (targeting Tyr421/Lys458 in spike protein), and inhibitors of the complement C5 protein (engaging Lys762) that protected cells from C5-induced lysis. It is likely that the dependence of the technique on context-dependent site-specific residue labelling reduces the possibility of off-target reactivity, and only sequences that place the warhead proximal to the nucleophilic site will be retained.

**Fig. 14:**
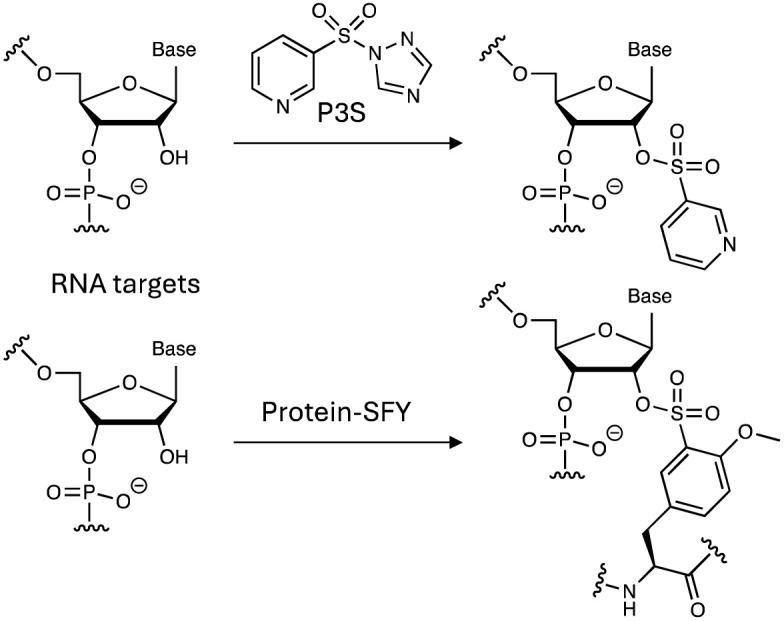
2′-OH modification of RNA using sulfonyl exchange chemistry.

Sulfonyl exchange chemistry also appears to be suitable for labelling the ribose 2′-OH of RNA (*cf.* DNA aptamers above) with utility in mapping RNA structures, as shown previously using acylation chemistry (a technique called SHAPE).^161^ A series of sulfonylating reagents, including SuDEx and SuTEx warheads, were tested for their ability to label RNA 2′-OH groups.^162^ Sulfonyl triazole P3S reacted with RNA (and not DNA) in high yield, and the sulfonate ester product possessed higher aqueous stability than acylated RNA, which facilitates structure analysis before hydrolysis loses signal (Fig. 14). P3S was shown to modify 2′-OH in a structure-sensitive manner on folded RNAs, and so the reagent could be used to complement SHAPE. This work also suggests that in the future covalent small molecule RNA-targeting drugs could be developed, and perhaps molecular glues that stick oligonucleotides to proteins.

2′-OH of RNA has also been targeted using encoded FSY and the more reactive *o*-sulfonyl fluoride–*O*-methyltyrosine (SFY, Fig. 12) tags in their binding proteins, enabling interrogation of protein–RNA interactions in cells (Fig. 14).^163^ FSY and SFY were incorporated into bacterial RNA binding protein Hfq, to site-specifically engage the ribose 2′-OH of bound RNAs that were identified using RT-qPCR. Genetically encoded SuFEx crosslinking has the potential to deliver higher sensitivity and lower side reactions than traditional photoaffinity labelling techniques such as CLIP-seq.

The interactions between proteins and carbohydrates play an important role in biology but have traditionally been difficult to interrogate. Protein–carbohydrate covalent crosslinking is hindered due to the relatively low nucleophilicity of the sugar hydroxyl groups. Therefore, the reactive SFY unnatural amino acid described above was encoded into proteins to enable crosslinking to their bound carbohydrates. As proof-of-concept, the transmembrane receptor Siglec-7, which is expressed on natural killer (NK) cells was armed with SFY and crosslinked its sialoglycan, G11 which is a tumour-associated carbohydrate antigen (FSY was unreactive).^164^ The technique not only has potential to elucidate fundamental aspects of glycobiology, but it has also revealed immunotherapeutic opportunities because the SFY-Siglec-7 was shown to enhance the killing of cancer cells by NK cells due to the upregulation of the sialoglycan in cancer.

## Future outlook

Sulfonyl exchange chemical biology has clearly advanced to the stage where covalent therapeutics that utilize this privileged chemistry are now within reach. I predict that within the next 5 years we will see covalent drug candidates armed with sulfonyl exchange warheads entering clinical trials. And within 10 years, I believe it is possible that the modality will realize its full therapeutic potential, where transformational drugs will reach the market that were rationally designed and developed to target residues beyond cysteine. The huge breadth of technologies developed using sulfonyl exchange chemistry in the last decade has been outstanding, and it will be exciting to see how these platforms continue to evolve. However, as would be expected from a somewhat nascent interdisciplinary field of research, there is room to grow and improve. By applying the learnings of the past (particularly in the field of cysteine-targeting), and exploring new scientific technologies, further innovations in covalent chemical biology will no doubt continue to proceed at a considerable pace, and below I highlight some possible areas of focus:

• Continue to apply novel chemoproteomic probes in new ways to answer biological/pharmacological questions. For instance, sulfonyl fluoride XO44 was originally developed to assess kinase inhibitor selectivity in cells by engaging the conserved lysine, but it has also been used to identify functional expression differences of kinases in cells,^78^ to identify ligandable tyrosines across diverse target space,^77^ and to reveal kinome plasticity across different species, which may impact species selection for preclinical safety studies of kinase inhibitors.^165^ XO44 was also used recently to show that palbociclib, a kinase inhibitor drug, binds to inactive CDK4 monomers and prevents interaction with p27.^166^ These studies illustrate the value that can be gleaned from diverse applications of covalent chemoproteomic probes.

• Further advance both the chemistry and proteomics of chemoproteomics. Greater warhead diversity will no doubt identify new ligandable sites, and new amino acid side chains, in proteins previously deemed undruggable.^167^ But innovations in MS proteomics techniques are also needed, such as the recent use of AzidoTMT to significantly expand multiplexing power, enabling the mapping of a set of 20 sulfonyl fluoride fragments employing DAS1 as the reporter.^168^

• Exploit chemoproteomics and structural bioinformatics to develop computational models, enhanced using machine learning methods, that help predict targetable sites, that will facilitate rational design and development of covalent probes (as being applied currently to the cysteinome).^169,170^

• Sulfonyl exchange chemoproteomics will likely contribute substantially to the development of transformational technologies that are required for initiatives such as target 2035, that has the ambitious objective of creating pharmacological tools for every human protein within the next 10 years.^171,172^ However, more studies are needed that convert ‘ligandable’ to ‘druggable’ site discovery using chemoproteomic probes bearing drug-compatible warheads incorporating privileged equilibrium binding motifs *i.e.* inverse drug discovery.

• Apply the privileged nature of sulfonyl exchange chemistry to the development of compound libraries that enable target identification from phenotypic screens, since protein labelling aids isolation and analysis. A recent report showed proof-of-concept using 32 sulfonyl fluoride probes screened against *Trypanosoma brucei*.^173^ This work could be extended to larger and more diverse libraries, exploring both the equilibrium binding and warhead motifs to enhance selectivity.

• The value of hit discovery and expansion using sulfonyl exchange technologies has been demonstrated and their development continues to evolve, and so these techniques should become broadly adopted. Some examples using sulfonyl exchange include the use of covalent docking and virtual screening^90^ (potentially enhanced using machine learning), direct-to-biology high-throughput synthesis and screening,^100^ and the use of covalent DEL libraries to expand covalency-first approaches to large diverse libraries.^48^

• Lead optimization of molecules possessing sulfonyl exchange electrophiles clearly needs more work, and this is where learning from the cysteine-targeting field could be leveraged. For instance, more studies should explore the metabolic pathways that reduce oral bioavailability of the covalent small molecules, which will help define a path to property-based optimization by the medicinal chemist.^94,101,174,175^ PK–PD modelling that has been tailored to acrylamide drugs can be applied to beyond-cysteine drugs too.^3,176^

• More safety profiling should be performed, including liver covalent binding burden studies using radiolabelled probes, as done for cysteine targeting drugs.^177^ Fluoride (SuFEx) and imidazole (SuDEx) are safe to very high concentrations in humans,^178,179^ but more studies are required to understand the toxic liabilities of other leaving groups.

• Synthetic chemistry: the progress made in sulfonyl exchange chemical biology would not have been possible without the innovative synthetic methods that have been developed over the last decade that have facilitated the preparation of a myriad of chemical probes. SuFEx preparative methodologies (reviewed elsewhere)^10,22–24^ have enhanced the covalent targeting toolkit and have been pivotal to the success of this field. Further innovations will include the development of chemoselective methods of their preparation that are resilient to a greater variety of functional groups,^79^ potentially allowing for late-stage functionalization, and the preparation of novel warheads that target even greater amino acid side chain diversity.

• Synthetic biology: genetic encoding of FSY and SFY to develop new protein-based therapeutics holds considerable promise, and additional unnatural amino acids bearing a variety of warheads to expand targetable biology can be envisaged. Exciting progress is being made in biomolecular engineering, including the stabilization of nanopore assemblies,^143^ and the controlled synthesis of neoproteins in cells that has the potential to rewire signalling circuits in cancer.^40^ SuFEx chemistry is also enabling the design of bacteria with synthetic cell walls,^180^ and bivalent chimeras are being used to effect new cell–cell proximity modalities.^181^

• Modality expansion enabled through sulfonyl exchange has been impressive, and the combination of different technologies seems to provide almost exponential diversity (*e.g.* combining covalent ligands with TPD and surface labelling methods, or covalent aptamers with autophagy-mediated degradation, and the like). Monovalent degraders that rely on covalent engagement of their targets will no doubt be an area of future study, although more work is needed to decipher the mechanisms at play, since many of these observations have been serendipitous thus far.^86,182^ The potential to drug other biomolecules, including RNA and carbohydrates is clearly an exciting avenue for future research and development. Sulfonyl exchange chemistry is also well-suited to the site-specific targeting of gain-of-tyrosine/lysine/histidine mutations in cancer, which appears to be an explored opportunity using sulfonyl exchange, though recent work has shown proof-of-concept through lysine targeting by salicylaldehydes.^183^

• Collaborations across industrial and academic sectors will be essential to ensure breakthroughs in the fundamental science of sulfonyl exchange chemistry and chemical biology are directed towards the development of impactful chemical probes, technologies and eventually, therapies. Team science, pooled resources, and integrated collaborations may help address current funding challenges at the private and public levels.

## Conclusions and reflections

Over 15 years ago, we created one of the first chemical biology groups in big pharma, with a mission to take ownership of a problem within the chemistry organization that has continued to plague drug discovery and has been the main reason for our collective failures – lack of efficacy of the candidate molecule in patients. Traditionally, discovery scientists had been so heavily focussed on moving high quality molecules into clinical trials, and satisfying metrics based on preclinical stage-gate transitions and the timely delivery of development candidates, that there was often a disconnect to the pivotal clinical proof-of-concept experiments where most attrition rests. At that time, cultural and organizational issues meant that there was a reluctance to ‘grasp the nettle’ with the conviction needed to stop preclinical work that would not be impactful in the long term, which would have been needed to redirect valuable resources towards the higher hanging fruit. Unfortunately, high value targets are very often difficult-to-drug and new modalities are required to expand druggability. To try and address this problem in an interdisciplinary manner, our chemical biology team started to consider covalent cysteine-targeting as a platform approach that could address challenging targets, and in those early stages our work revealed that JAK3 and TAK1 were clearly actionable targets for the treatment of inflammatory disorders.^184,185^ We then evolved our ‘covalent chemical biology platform’ idea to include residues beyond cysteine, for the reasons described throughout this Perspective. Our efforts were inspired by the pioneers of sulfonyl exchange chemistry, including Bernard Baker and Roberta Colman, whose seminal contributions to the field many years previously deserve more recognition than they receive.^13,16^ Our work coincided with that of Sharpless and Taunton (the latter a collaborator), and later efforts such as those of the Wang group have been instrumental to advance this exciting area of covalent chemical biology, across both small and large molecule modalities.

Unequivocally, there are a myriad of opportunities afforded by the uniquely privileged nature of sulfonyl exchange chemical biology. The breadth of covalent therapeutic modalities and technologies using sulfonyl exchange electrophiles is remarkable, which will allow many targets previously deemed undruggable to be addressed for the first time. Indeed, several new biotech companies are currently employing covalent platform chemistries, including sulfonyl exchange warheads, to target protein residues beyond cysteine. These efforts span small molecule TCIs and protein therapeutics, and covalent peptides appear to be ripe for investment too due to the scientific progress being made here. Our own work that exemplifies the drug-compatible nature of certain emerging sulfonyl exchange warheads suggests that a key change in this area will be the evolution of the field from useful proof-of-concept chemical biology probes and technologies to the design of drug candidates that address compelling therapeutic targets. When I write the next update of this field in 10 years' time, I hope to describe breakthroughs in covalent chemical biology that have clearly advanced the development of therapies with transformational efficacy.

## Conflicts of interest

L. H. J. is a co-founder of Anvia, an advisor to ONO Pharma, Merck KGaA, Rapafusyn Pharmaceuticals, Matchpoint Therapeutics, Lime Therapeutics, Immunovalence, and Belharra Therapeutics, and holds equity in Hyku Biosciences and Rapafusyn Pharmaceuticals. L. H. J previously received funding from Deerfield at the Center for Protein Degradation at DFCI.

## Data Availability

No primary research results, software or code have been included and no new data were generated or analysed as part of this review.

## References

[cit1] Bunnage M. E., Chekler E. L., Jones L. H. (2013). Nat. Chem. Biol..

[cit2] Stepan A. F., Walker D. P., Bauman J., Price D. A., Baillie T. A., Kalgutkar A. S., Aleo M. D. (2011). Chem. Res. Toxicol..

[cit3] Jones L. H. (2021). Annu. Rep. Med. Chem..

[cit4] Lonsdale R., Ward R. A. (2018). Chem. Soc. Rev..

[cit5] Daryaee F., Zhang Z., Gogarty K. R., Li Y., Merino J., Fisher S. L., Tonge P. J. (2017). Chem. Sci..

[cit6] Strelow J. M. (2017). SLAS Discov..

[cit7] Leishman D. J., Brimecombe J., Crumb W., Hebeisen S., Jenkinson S., Kilfoil P. J., Matsukawa H., Melliti K., Qu Y. (2024). J. Pharmacol. Toxicol. Methods.

[cit8] Bunnage M. E., Chekler E. L., Jones L. H. (2013). Nat. Chem. Biol..

[cit9] Jones L. H. , Kenakin T., in Comprehensive Pharmacology, Elsevier, 2022, pp. 476–497

[cit10] Huang H., Jones L. H. (2023). Expet Opin. Drug Discov..

[cit11] Khazanov N. A., Carlson H. A. (2013). PLoS Comput. Biol..

[cit12] Baker B. R. (1959). Cancer Chemother. Rep..

[cit13] Baker B. R. (1970). Annu. Rev. Pharmacol..

[cit14] Gold A. M., Fahrney D. (1964). Biochemistry.

[cit15] GOLD A. M. (1965). Biochemistry.

[cit16] Colman R. F. (1983). Annu. Rev. Biochem..

[cit17] Pal P. K., Wechter W. J., Colman R. F. (1975). J. Biol. Chem..

[cit18] Zhao Q., Ouyang X., Wan X., Gajiwala K. S., Kath J. C., Jones L. H., Burlingame A. L., Taunton J. (2017). J. Am. Chem. Soc..

[cit19] Narayanan A., Jones L. H. (2015). Chem. Sci..

[cit20] Hett E. C., Xu H., Geoghegan K. F., Gopalsamy A., Kyne R. E., Menard C. A., Narayanan A., Parikh M. D., Liu S., Roberts L., Robinson R. P., Tones M. A., Jones L. H. (2015). ACS Chem. Biol..

[cit21] Jones L. H. , Presented in part at the 248th ACS National Meeting, San Francisco, 2014

[cit22] Dong J., Krasnova L., Finn M. G., Sharpless K. B. (2014). Angew Chem. Int. Ed. Engl..

[cit23] Lou T. S., Willis M. C. (2022). Nat. Rev. Chem.

[cit24] Barrow A. S., Smedley C. J., Zheng Q., Li S., Dong J., Moses J. E. (2019). Chem. Soc. Rev..

[cit25] Steinkopf W. (1927). J. Prakt. Chem..

[cit26] Davies W., Dick J. H. J. Chem. Soc..

[cit27] Narayan S., Muldoon J., Finn M. G., Fokin V. V., Kolb H. C., Sharpless K. B. (2005). Angew Chem. Int. Ed. Engl..

[cit28] Brown D. J., Hoskins J. A. (1972). J. Chem. Soc., Perkin 1.

[cit29] Wang L., Cornella J. (2020). Angew Chem. Int. Ed. Engl..

[cit30] Wright S. W., Hallstrom K. N. (2006). J. Org. Chem..

[cit31] Jiang Y., Alharbi N. S., Sun B., Qin H. L. (2019). RSC Adv..

[cit32] Brouwer A. J., Ceylan T., Jonker A. M., van der Linden T., Liskamp R. M. (2011). Bioorg. Med. Chem..

[cit33] Davies A. T., Curto J. M., Bagley S. W., Willis M. C. (2017). Chem. Sci..

[cit34] Tribby A. L., Rodríguez I., Shariffudin S., Ball N. D. (2017). J. Org. Chem..

[cit35] Deeming A. S., Russell C. J., Willis M. C. (2016). Angew Chem. Int. Ed. Engl..

[cit36] Chen Y., Willis M. C. (2017). Chem. Sci..

[cit37] Hansen T. N., Segundo M. S., Mergel A. M., Olsen C. A. (2025). Trends Chem..

[cit38] Jones L. H., Xu H., Fadeyi O. O. (2019). Methods Enzymol..

[cit39] Jones L. H., Kelly J. W. (2020). RSC Med. Chem..

[cit40] Jones L. H. (2024). RSC Med. Chem..

[cit41] Ran X., Gestwicki J. E. (2018). Curr. Opin. Chem. Biol..

[cit42] Liu S., Dakin L. A., Xing L., Withka J. M., Sahasrabudhe P. V., Li W., Banker M. E., Balbo P., Shanker S., Chrunyk B. A., Guo Z., Chen J. M., Young J. A., Bai G., Starr J. T., Wright S. W., Bussenius J., Tan S., Gopalsamy A., Lefker B. A., Vincent F., Jones L. H., Xu H., Hoth L. R., Geoghegan K. F., Qiu X., Bunnage M. E., Thorarensen A. (2016). Sci. Rep..

[cit43] Mukherjee H., Su N., Belmonte M. A., Hargreaves D., Patel J., Tentarelli S., Aquila B., Grimster N. P. (2019). Bioorg. Med. Chem. Lett..

[cit44] Kerres N., Steurer S., Schlager S., Bader G., Berger H., Caligiuri M., Dank C., Engen J. R., Ettmayer P., Fischerauer B., Flotzinger G., Gerlach D., Gerstberger T., Gmaschitz T., Greb P., Han B., Heyes E., Iacob R. E., Kessler D., Kölle H., Lamarre L., Lancia D. R., Lucas S., Mayer M., Mayr K., Mischerikow N., Mück K., Peinsipp C., Petermann O., Reiser U., Rudolph D., Rumpel K., Salomon C., Scharn D., Schnitzer R., Schrenk A., Schweifer N., Thompson D., Traxler E., Varecka R., Voss T., Weiss-Puxbaum A., Winkler S., Zheng X., Zoephel A., Kraut N., McConnell D., Pearson M., Koegl M. (2017). Cell Rep..

[cit45] Teng M., Ficarro S. B., Yoon H., Che J., Zhou J., Fischer E. S., Marto J. A., Zhang T., Gray N. S. (2020). ACS Med. Chem. Lett..

[cit46] Bum-Erdene K., Liu D., Gonzalez-Gutierrez G., Ghozayel M. K., Xu D., Meroueh S. O. (2020). Proc. Natl. Acad. Sci. U. S. A..

[cit47] Landgraf A. D., Yeh I. J., Ghozayel M. K., Bum-Erdene K., Gonzalez-Gutierrez G., Meroueh S. O. (2023). ChemMedChem.

[cit48] Jiang L., Liu S., Jia X., Gong Q., Wen X., Lu W., Yang J., Wu X., Wang X., Suo Y., Li Y., Uesugi M., Qu Z. B., Tan M., Lu X., Zhou L. (2023). J. Am. Chem. Soc..

[cit49] Hatcher J. M., Wu G., Zeng C., Zhu J., Meng F., Patel S., Wang W., Ficarro S. B., Leggett A. L., Powell C. E., Marto J. A., Zhang K., Ki Ngo J. C., Fu X. D., Zhang T., Gray N. S. (2018). Cell Chem. Biol..

[cit50] Jones L. H. (2018). Angew Chem. Int. Ed. Engl..

[cit51] Cruite J. T., Dann G. P., Che J., Donovan K. A., Ferrao S., Ficarro S. B., Fischer E. S., Gray N. S., Huerta F., Kong N. R., Liu H., Marto J. A., Metivier R. J., Nowak R. P., Zerfas B. L., Jones L. H. (2022). RSC Chem. Biol..

[cit52] Jones L. H. (2018). ACS Med. Chem. Lett..

[cit53] Wang Z., Zhu B., Jiang F., Chen X., Wang G., Ding N., Song S., Xu X., Zhang W. (2024). Bioorg. Med. Chem..

[cit54] Craig A., Kogler J., Laube M., Ullrich M., Donat C. K., Wodtke R., Kopka K., Stadlbauer S. (2023). Pharmaceutics.

[cit55] Deng X., Zhu X. (2023). ACS Omega.

[cit56] Zheng Q., Xu H., Wang H., Du W. H., Wang N., Xiong H., Gu Y., Noodleman L., Sharpless K. B., Yang G., Wu P. (2021). J. Am. Chem. Soc..

[cit57] Bolding J. E., Martín-Gago P., Rajabi N., Gamon L. F., Hansen T. N., Bartling C. R. O., Strømgaard K., Davies M. J., Olsen C. A. (2022). Angew Chem. Int. Ed. Engl..

[cit58] McFadden W. M., Casey-Moore M. C., Bare G. A. L., Kirby K. A., Wen X., Li G., Wang H., Slack R. L., Snyder A. A., Lorson Z. C., Kaufman I. L., Cilento M. E., Tedbury P. R., Gembicky M., Olson A. J., Torbett B. E., Sharpless K. B., Sarafianos S. G. (2024). Cell Chem. Biol..

[cit59] Wang S., Faucher F. F., Bertolini M., Kim H., Yu B., Cao L., Roeltgen K., Lovell S., Shanker V., Boyd S. D., Wang L., Bartenschlager R., Bogyo M. (2025). J. Am. Chem. Soc..

[cit60] Beauglehole A. R., Baker S. P., Scammells P. J. (2000). J. Med. Chem..

[cit61] Glukhova A., Thal D. M., Nguyen A. T., Vecchio E. A., Jörg M., Scammells P. J., May L. T., Sexton P. M., Christopoulos A. (2017). Cell.

[cit62] Yang X., van Veldhoven J. P. D., Offringa J., Kuiper B. J., Lenselink E. B., Heitman L. H., van der Es D., IJzerman A. P. (2019). J. Med. Chem..

[cit63] Yang X., Dilweg M. A., Osemwengie D., Burggraaff L., van der Es D., Heitman L. H., IJzerman A. P. (2020). Biochem. Pharmacol..

[cit64] Payne C. M., Baltos J. A., Langiu M., Sinh Lu C., Tyndall J. D. A., Gregory K. J., May L. T., Vernall A. J. (2024). Chembiochem.

[cit65] Yang X., Dong G., Michiels T. J., Lenselink E. B., Heitman L., Louvel J., IJzerman A. P. (2016). Purinergic Signal..

[cit66] Grimster N. P., Connelly S., Baranczak A., Dong J., Krasnova L. B., Sharpless K. B., Powers E. T., Wilson I. A., Kelly J. W. (2013). J. Am. Chem. Soc..

[cit67] Baranczak A., Liu Y., Connelly S., Du W. G., Greiner E. R., Genereux J. C., Wiseman R. L., Eisele Y. S., Bradbury N. C., Dong J., Noodleman L., Sharpless K. B., Wilson I. A., Encalada S. E., Kelly J. W. (2015). J. Am. Chem. Soc..

[cit68] Garner M. H., Bogardt R. A., Gurd F. R. (1975). J. Biol. Chem..

[cit69] Liu Q., Sabnis Y., Zhao Z., Zhang T., Buhrlage S. J., Jones L. H., Gray N. S. (2013). Chem. Biol..

[cit70] Dalton S. E., Dittus L., Thomas D. A., Convery M. A., Nunes J., Bush J. T., Evans J. P., Werner T., Bantscheff M., Murphy J. A., Campos S. (2018). J. Am. Chem. Soc..

[cit71] Liu R., Yue Z., Tsai C. C., Shen J. (2019). J. Am. Chem. Soc..

[cit72] Hanoulle X., van Damme J., Staes A., Martens L., Goethals M., Vandekerckhove J., Gevaert K. (2006). J. Proteome Res..

[cit73] Jones L. H. (2015). Future Med. Chem..

[cit74] Arafet K., Scalvini L., Galvani F., Martí S., Moliner V., Mor M., Lodola A. (2023). J. Chem. Inf. Model..

[cit75] Freeman-Cook K., Hoffman R. L., Miller N., Almaden J., Chionis J., Zhang Q., Eisele K., Liu C., Zhang C., Huser N., Nguyen L., Costa-Jones C., Niessen S., Carelli J., Lapek J., Weinrich S. L., Wei P., McMillan E., Wilson E., Wang T. S., McTigue M., Ferre R. A., He Y. A., Ninkovic S., Behenna D., Tran K. T., Sutton S., Nagata A., Ornelas M. A., Kephart S. E., Zehnder L. R., Murray B., Xu M., Solowiej J. E., Visswanathan R., Boras B., Looper D., Lee N., Bienkowska J. R., Zhu Z., Kan Z., Ding Y., Mu X. J., Oderup C., Salek-Ardakani S., White M. A., VanArsdale T., Dann S. G. (2021). Cancer Cell.

[cit76] Lou K., Wassarman D. R., Yang T., Paung Y., Zhang Z., O'Loughlin T. A., Moore M. K., Egan R. K., Greninger P., Benes C. H., Seeliger M. A., Taunton J., Gilbert L. A., Shokat K. M. (2022). Science.

[cit77] van Bergen W., Nederstigt A. E., Heck A. J. R., Baggelaar M. P. (2025). Mol. Cell. Proteomics.

[cit78] Leung C. O. N., Yang Y., Leung R. W. H., So K. K. H., Guo H. J., Lei M. M. L., Muliawan G. K., Gao Y., Yu Q. Q., Yun J. P., Ma S., Zhao Q., Lee T. K. W. (2023). Nat. Commun..

[cit79] Fadeyi O., Parikh M. D., Chen M. Z., Kyne R. E., Taylor A. P., O'Doherty I., Kaiser S. E., Barbas S., Niessen S., Shi M., Weinrich S. L., Kath J. C., Jones L. H., Robinson R. P. (2016). Chembiochem.

[cit80] Ferlenghi F., Scalvini L., Vacondio F., Castelli R., Bozza N., Marseglia G., Rivara S., Lodola A., La Monica S., Minari R., Petronini P. G., Alfieri R., Tiseo M., Mor M. (2021). Eur. J. Med. Chem..

[cit81] Gilbert K. E., Vuorinen A., Aatkar A., Pogány P., Pettinger J., Grant E. K., Kirkpatrick J. M., Rittinger K., House D., Burley G. A., Bush J. T. (2023). ACS Chem. Biol..

[cit82] Tang G., Wang W., Zhu C., Huang H., Chen P., Wang X., Xu M., Sun J., Zhang C. J., Xiao Q., Gao L., Zhang Z. M., Yao S. Q. (2024). Angew Chem. Int. Ed. Engl..

[cit83] Tang G., Wang W., Wang X., Ding K., Ngan S. C., Chen J. Y., Sze S. K., Gao L., Yuan P., Lu X., Yao S. Q. (2023). Eur. J. Med. Chem..

[cit84] Cosgrove B., Grant E. K., Bertrand S., Down K. D., Somers D. O., P Evans J., Tomkinson N. C. O., Barker M. D. (2023). RSC Chem. Biol..

[cit85] Pettinger J., Carter M., Jones K., Cheeseman M. D. (2019). J. Med. Chem..

[cit86] Cuesta A., Wan X., Burlingame A. L., Taunton J. (2020). J. Am. Chem. Soc..

[cit87] Jones L. H. (2018). Cell Chem. Biol..

[cit88] Alboreggia G., Udompholkul P., Rodriguez I., Blaha G., Pellecchia M. (2024). Proc. Natl. Acad. Sci. U. S. A..

[cit89] Zammit C. M., Nadel C. M., Lin Y., Koirala S., Potts P. R., Nomura D. K. (2025). bioRxiv.

[cit90] Wan X., Yang T., Cuesta A., Pang X., Balius T. E., Irwin J. J., Shoichet B. K., Taunton J. (2020). J. Am. Chem. Soc..

[cit91] Marcotrigiano J., Gingras A. C., Sonenberg N., Burley S. K. (1997). Cell.

[cit92] Ding J., Li G., Liu H., Liu L., Lin Y., Gao J., Zhou G., Shen L., Zhao M., Yu Y., Guo W., Hommel U., Ottl J., Blank J., Aubin N., Wei Y., He H., Sage D. R., Atadja P. W., Li E., Jain R. K., Tallarico J. A., Canham S. M., Chiang Y. L., Wang H. (2023). ACS Chem. Biol..

[cit93] Chen Y., Craven G. B., Kamber R. A., Cuesta A., Zhersh S., Moroz Y. S., Bassik M. C., Taunton J. (2023). Nat. Chem..

[cit94] Baggio C., Udompholkul P., Gambini L., Salem A. F., Jossart J., Perry J. J. P., Pellecchia M. (2019). J. Med. Chem..

[cit95] Watson E. R., Novick S., Matyskiela M. E., Chamberlain P. P., H de la Peña A., Zhu J., Tran E., Griffin P. R., Wertz I. E., Lander G. C. (2022). Science.

[cit96] Cruite J. T., Dann G. P., Che J., Donovan K. A., Ferrao S., Fischer E. S., Gray N. S., Huerta F., Kong N. R., Liu H., Marto J., Metivier R. J., Nowak R. P., Zerfas B. L., Jones L. H. (2022). RSC Chem. Biol..

[cit97] Dann G. P., Liu H., Nowak R. P., Jones L. H. (2023). Methods Enzymol..

[cit98] Kong N. R., Liu H., Che J., Jones L. H. (2021). ACS Med. Chem. Lett..

[cit99] Cruite J. T., Nowak R. P., Donovan K. A., Ficarro S. B., Huang H., Liu H., Liu Y., Marto J. A., Metivier R. J., Fischer E. S., Jones L. H. (2023). ACS Med. Chem. Lett..

[cit100] Aatkar A., Vuorinen A., Longfield O. E., Gilbert K., Peltier-Heap R., Wagner C. D., Zappacosta F., Rittinger K., Chung C. W., House D., Tomkinson N. C. O., Bush J. T. (2023). ACS Chem. Biol..

[cit101] Che J., Jones L. H. (2022). RSC Med. Chem..

[cit102] Pahari S., Sun L., Alexov E. (2019). Database.

[cit103] Ribeiro A. J. M., Tyzack J. D., Borkakoti N., Holliday G. L., Thornton J. M. (2020). J. Biol. Chem..

[cit104] Soga S., Shirai H., Kobori M., Hirayama N. (2007). J. Chem. Inf. Model..

[cit105] Powers J. C., Asgian J. L., Ekici O. D., James K. E. (2002). Chem. Rev..

[cit106] Rawlings N. D., Waller M., Barrett A. J., Bateman A. (2014). Nucleic Acids Res..

[cit107] Artschwager R., Ward D. J., Gannon S., Brouwer A. J., van de Langemheen H., Kowalski H., Liskamp R. M. J. (2018). J. Med. Chem..

[cit108] Zheng Q., Woehl J. L., Kitamura S., Santos-Martins D., Smedley C. J., Li G., Forli S., Moses J. E., Wolan D. W., Sharpless K. B. (2019). Proc. Natl. Acad. Sci. U. S. A..

[cit109] Du S., Kretsch R. C., Parres-Gold J., Pieri E., Cruzeiro V. W. D., Zhu M., Pinney M. M., Yabukarski F., Schwans J. P., Martínez T. J., Herschlag D. (2025). Science.

[cit110] Fadeyi O. O., Hoth L. R., Choi C., Feng X., Gopalsamy A., Hett E. C., Kyne R. E., Robinson R. P., Jones L. H. (2017). ACS Chem. Biol..

[cit111] Roth G. J., Machuga E. T., Ozols J. (1983). Biochemistry.

[cit112] Jones L. H. (2020). RSC Chem. Biol..

[cit113] Yang B., Wang N., Schnier P. D., Zheng F., Zhu H., Polizzi N. F., Ittuveetil A., Saikam V., DeGrado W. F., Wang Q., Wang P. G., Wang L. (2019). J. Am. Chem. Soc..

[cit114] Shah R. R., De Vita E., Sathyamurthi P. S., Conole D., Zhang X., Fellows E., Dickinson E. R., Fleites C. M., Queisser M. A., Harling J. D., Tate E. W. (2024). J. Med. Chem..

[cit115] Doornbos M. L. J., Wang X., Vermond S. C., Peeters L., Pérez-Benito L., Trabanco A. A., Lavreysen H., Cid J. M., Heitman L. H., Tresadern G., IJzerman A. P. (2019). J. Med. Chem..

[cit116] Liu Y., Nowak R. P., Che J., Donovan K. A., Huerta F., Liu H., Metivier R. J., Fischer E. S., Jones L. H. (2024). RSC Med. Chem..

[cit117] Gechijian L. N., Muncipinto G., Rettenmaier T. J., Labenski M. T., Rusu V., Rosskamp L., Conway L., van Kalken D., Gross L., Iantosca G., Crotty W., Mathis R., Park H., Rabin B., Westgate C., Lyons M., Deshusses C., Brandon N., Brown D. G., Blanchette H. S., Pullen N., Jones L. H., Barrish J. C. (2024). ACS Chem. Biol..

[cit118] Cravatt B. F., Wright A. T., Kozarich J. W. (2008). Annu. Rev. Biochem..

[cit119] Sanman L. E., Bogyo M. (2014). Annu. Rev. Biochem..

[cit120] Jones L. H. , Gordon Research Conference, Andover NH, 2019

[cit121] Gu C., Shannon D. A., Colby T., Wang Z., Shabab M., Kumari S., Villamor J. G., McLaughlin C. J., Weerapana E., Kaiser M., Cravatt B. F., van der Hoorn R. A. (2013). Chem. Biol..

[cit122] Hahm H. S., Toroitich E. K., Borne A. L., Brulet J. W., Libby A. H., Yuan K., Ware T. B., McCloud R. L., Ciancone A. M., Hsu K. L. (2020). Nat. Chem. Biol..

[cit123] Brulet J. W., Borne A. L., Yuan K., Libby A. H., Hsu K. L. (2020). J. Am. Chem. Soc..

[cit124] Ciancone A. M., Hosseinibarkooie S., Bai D. L., Borne A. L., Ferris H. A., Hsu K. L. (2022). Cell Chem. Biol..

[cit125] Huang T., Hosseinibarkooie S., Borne A. L., Granade M. E., Brulet J. W., Harris T. E., Ferris H. A., Hsu K. L. (2021). Chem. Sci..

[cit126] Toroitich E. K., Ciancone A. M., Hahm H. S., Brodowski S. M., Libby A. H., Hsu K. L. (2021). Chembiochem.

[cit127] Ciancone A. M., Seo K. W., Chen M., Borne A. L., Libby A. H., Bai D. L., Kleiner R. E., Hsu K. L. (2023). J. Am. Chem. Soc..

[cit128] Mendez R., Shaikh M., Lemke M. C., Yuan K., Libby A. H., Bai D. L., Ross M. M., Harris T. E., Hsu K. L. (2023). RSC Chem. Biol..

[cit129] McCloud R. L., Yuan K., Mahoney K. E., Bai D. L., Shabanowitz J., Ross M. M., Hunt D. F., Hsu K. L. (2021). Anal. Chem..

[cit130] Brighty G. J., Botham R. C., Li S., Nelson L., Mortenson D. E., Li G., Morisseau C., Wang H., Hammock B. D., Sharpless K. B., Kelly J. W. (2020). Nat. Chem..

[cit131] Justin Grams R., Yuan K., Founds M. W., Ware M. L., Pilar M. G., Hsu K. L. (2024). Chembiochem.

[cit132] Yang B., Wu H., Schnier P. D., Liu Y., Liu J., Wang N., DeGrado W. F., Wang L. (2018). Proc. Natl. Acad. Sci. U. S. A..

[cit133] Nowak R. P., Ragosta L., Huerta F., Liu H., Ficarro S. B., Cruite J. T., Metivier R. J., Donovan K. A., Marto J. A., Fischer E. S., Zerfas B. L., Jones L. H. (2023). RSC Chem. Biol..

[cit134] Grimster N. P. (2021). RSC Med. Chem..

[cit135] Carvalho L. A. R., Sousa B. B., Zaidman D., Kiely-Collins H., Bernardes G. J. L. (2024). Chembiochem.

[cit136] Matsuo K., Nishikawa Y., Masuda M., Hamachi I. (2018). Angew Chem. Int. Ed. Engl..

[cit137] Luo Q., Wang Y., Hou Z., Liang H., Tu L., Xing Y., Wan C., Liu J., Wang R., Zhu L., Han W., Wu J., Lu F., Yin F., Li Z. (2024). Chem. Commun..

[cit138] Berdan V. Y., Klauser P. C., Wang L. (2021). Bioorg. Med. Chem..

[cit139] Hoppmann C., Wang L. (2016). Chem. Commun..

[cit140] Stewart M. L., Fire E., Keating A. E., Walensky L. D. (2010). Nat. Chem. Biol..

[cit141] Alboreggia G., Udompholkul P., Atienza E. L., Muzzarelli K., Assar Z., Pellecchia M. (2024). J. Med. Chem..

[cit142] Zhang M. Y., Yang H., Ortiz G., Trnka M. J., Petronikolou N., Burlingame A. L., DeGrado W. F., Fujimori D. G. (2022). Chem. Sci..

[cit143] Schnaider L., Tan S., Singh P. R., Capuano F., Scott A. J., Hambley R., Lu L., Yang H., Wallace E. J., Jo H., DeGrado W. F. (2024). J. Am. Chem. Soc..

[cit144] McCann H. M., Lake B. P. M., Hoffman K. S., Davola M. E., Mossman K. L., Rullo A. F. (2022). ACS Chem. Biol..

[cit145] Serniuck N. J., Kapcan E., Moogk D., Moore A. E., Lake B. P. M., Denisova G., Hammill J. A., Bramson J. L., Rullo A. F. (2024). Mol. Ther. Oncol..

[cit146] Wang N., Yang B., Fu C., Zhu H., Zheng F., Kobayashi T., Liu J., Li S., Ma C., Wang P. G., Wang Q., Wang L. (2018). J. Am. Chem. Soc..

[cit147] Liu J., Cao L., Klauser P. C., Cheng R., Berdan V. Y., Sun W., Wang N., Ghelichkhani F., Yu B., Rozovsky S., Wang L. (2021). J. Am. Chem. Soc..

[cit148] Li Q., Chen Q., Klauser P. C., Li M., Zheng F., Wang N., Li X., Zhang Q., Fu X., Wang Q., Xu Y., Wang L. (2020). Cell.

[cit149] Yu B., Li S., Tabata T., Wang N., Cao L., Kumar G. R., Sun W., Liu J., Ott M., Wang L. (2022). Chem.

[cit150] Zhang H., Han Y., Yang Y., Lin F., Li K., Kong L., Liu H., Dang Y., Lin J., Chen P. R. (2021). J. Am. Chem. Soc..

[cit151] Banik S. M., Pedram K., Wisnovsky S., Ahn G., Riley N. M., Bertozzi C. R. (2020). Nature.

[cit152] Cotton A. D., Nguyen D. P., Gramespacher J. A., Seiple I. B., Wells J. A. (2021). J. Am. Chem. Soc..

[cit153] Yu B., Cao L., Li S., Klauser P. C., Wang L. (2023). Chem. Sci..

[cit154] Xiao Y., He Z., Li W., Chen D., Niu X., Yang X., Zeng W., Wang M., Qian Y., Su Y., Luo F., Chen G., Liu J., Sui X., Zhou X., Gao Y. (2025). Nat. Commun..

[cit155] Xiang S., Zhu C., Zhou Y., Wu W., Zhang Y., Chen C., Wang F. (2024). ACS Chem. Biol..

[cit156] Klauser P. C., Chopra S., Cao L., Bobba K. N., Yu B., Seo Y., Chan E., Flavell R. R., Evans M. J., Wang L. (2023). ACS Cent. Sci..

[cit157] Cui X. Y., Li Z., Kong Z., Liu Y., Meng H., Wen Z., Wang C., Chen J., Xu M., Li Y., Gao J., Zhu W., Hao Z., Huo L., Liu S., Yang Z., Liu Z. (2024). Nature.

[cit158] Tabuchi Y., Yang J., Taki M. (2021). Chem. Commun..

[cit159] Shi Y., Yun Y., Wang R., Liu Z., Wu Z., Xiang Y., Zhang J. (2025). Angew Chem. Int. Ed. Engl..

[cit160] Qin Z., Zhang K., He P., Zhang X., Xie M., Fu Y., Gu C., Zhu Y., Tong A., Wei H., Zhang C., Xiang Y. (2023). Nat. Chem..

[cit161] Merino E. J., Wilkinson K. A., Coughlan J. L., Weeks K. M. (2005). J. Am. Chem. Soc..

[cit162] Chatterjee S., Shioi R., Kool E. T. (2023). ACS Cent. Sci..

[cit163] Sun W., Wang N., Liu H., Yu B., Jin L., Ren X., Shen Y., Wang L. (2023). Nat. Chem..

[cit164] Li S., Wang N., Yu B., Sun W., Wang L. (2023). Nat. Chem..

[cit165] Marholz L. J., Federspiel J. D., Suh H., Fernandez Ocana M. (2021). J. Proteome Res..

[cit166] Guiley K. Z., Stevenson J. W., Lou K., Barkovich K. J., Kumarasamy V., Wijeratne T. U., Bunch K. L., Tripathi S., Knudsen E. S., Witkiewicz A. K., Shokat K. M., Rubin S. M. (2019). Science.

[cit167] Abbasov M. E., Kavanagh M. E., Ichu T. A., Lazear M. R., Tao Y., Crowley V. M., Am Ende C. W., Hacker S. M., Ho J., Dix M. M., Suciu R., Hayward M. M., Kiessling L. L., Cravatt B. F. (2021). Nat. Chem..

[cit168] Ma T. P., Izrael-Tomasevic A., Mroue R., Budayeva H., Malhotra S., Raisner R., Evangelista M., Rose C. M., Kirkpatrick D. S., Yu K. (2023). J. Proteome Res..

[cit169] Liu R., Clayton J., Shen M., Bhatnagar S., Shen J. (2024). JACS Au.

[cit170] Reimer B. M., Awoonor-Williams E., Golosov A. A., Hornak V. (2025). J. Chem. Inf. Model..

[cit171] Müller S., Ackloo S., Al Chawaf A., Al-Lazikani B., Antolin A., Baell J. B., Beck H., Beedie S., Betz U. A. K., Bezerra G. A., Brennan P. E., Brown D., Brown P. J., Bullock A. N., Carter A. J., Chaikuad A., Chaineau M., Ciulli A., Collins I., Dreher J., Drewry D., Edfeldt K., Edwards A. M., Egner U., Frye S. V., Fuchs S. M., Hall M. D., Hartung I. V., Hillisch A., Hitchcock S. H., Homan E., Kannan N., Kiefer J. R., Knapp S., Kostic M., Kubicek S., Leach A. R., Lindemann S., Marsden B. D., Matsui H., Meier J. L., Merk D., Michel M., Morgan M. R., Mueller-Fahrnow A., Owen D. R., Perry B. G., Rosenberg S. H., Saikatendu K. S., Schapira M., Scholten C., Sharma S., Simeonov A., Sundström M., Superti-Furga G., Todd M. H., Tredup C., Vedadi M., von Delft F., Willson T. M., Winter G. E., Workman P., Arrowsmith C. H. (2022). RSC Med. Chem..

[cit172] Ackloo S., Antolin A. A., Bartolome J. M., Beck H., Bullock A., Betz U. A. K., Böttcher J., Brown P. J., Chaturvedi M., Crisp A., Daniels D., Dreher J., Edfeldt K., Edwards A. M., Egner U., Elkins J., Fischer C., Glendorf T., Goldberg S., Hartung I. V., Hillisch A., Homan E., Knapp S., Köster M., Krämer O., Llaveria J., Lessel U., Lindemann S., Linderoth L., Matsui H., Michel M., Montel F., Mueller-Fahrnow A., Müller S., Owen D. R., Saikatendu K. S., Santhakumar V., Sanderson W., Scholten C., Schapira M., Sharma S., Shireman B., Sundström M., Todd M. H., Tredup C., Venable J., Willson T. M., Arrowsmith C. H. (2023). RSC Med. Chem..

[cit173] Mantilla B. S., White J. S., Mosedale W. R. T., Gomm A., Nelson A., Smith T. K., Wright M. H. (2024). Commun. Chem..

[cit174] Mukherjee H., Debreczeni J., Breed J., Tentarelli S., Aquila B., Dowling J. E., Whitty A., Grimster N. P. (2017). Org. Biomol. Chem..

[cit175] King A. T., Matesic L., Keaveney S. T., Jamie J. F. (2023). Mol. Pharm..

[cit176] Leung L., Yang X., Strelevitz T. J., Montgomery J., Brown M. F., Zientek M. A., Banfield C., Gilbert A. M., Thorarensen A., Dowty M. E. (2017). Drug Metab. Dispos..

[cit177] Dahal U. P., Obach R. S., Gilbert A. M. (2013). Chem. Res. Toxicol..

[cit178] Guth S., Hüser S., Roth A., Degen G., Diel P., Edlund K., Eisenbrand G., Engel K. H., Epe B., Grune T., Heinz V., Henle T., Humpf H. U., Jäger H., Joost H. G., Kulling S. E., Lampen A., Mally A., Marchan R., Marko D., Mühle E., Nitsche M. A., Röhrdanz E., Stadler R., van Thriel C., Vieths S., Vogel R. F., Wascher E., Watzl C., Nöthlings U., Hengstler J. G. (2020). Arch. Toxicol..

[cit179] BASF, CLH report for imidazole, https://echa.europa.eu/documents/10162/9a2b728c-34ac-b4a5-d295-a1ae1b42f613

[cit180] Dik D. A., Zhang N., Chen J. S., Webb B., Schultz P. G. (2020). J. Am. Chem. Soc..

[cit181] Kapcan E., Krygier K., da Luz M., Serniuck N. J., Zhang A., Bramson J., Rullo A. F. (2025). Nat. Commun..

[cit182] Jones L. H. (2018). Cell Chem. Biol..

[cit183] Craven G. B., Chu H., Sun J. D., Carelli J. D., Coyne B., Chen H., Chen Y., Ma X., Das S., Kong W., Zajdlik A. D., Yang K. S., Reisberg S. H., Thompson P. A., Lipford J. R., Taunton J. (2025). Nature.

[cit184] Telliez J. B., Dowty M. E., Wang L., Jussif J., Lin T., Li L., Moy E., Balbo P., Li W., Zhao Y., Crouse K., Dickinson C., Symanowicz P., Hegen M., Banker M. E., Vincent F., Unwalla R., Liang S., Gilbert A. M., Brown M. F., Hayward M., Montgomery J., Yang X., Bauman J., Trujillo J. I., Casimiro-Garcia A., Vajdos F. F., Leung L., Geoghegan K. F., Quazi A., Xuan D., Jones L., Hett E., Wright K., Clark J. D., Thorarensen A. (2016). ACS Chem. Biol..

[cit185] Kilty I., Green M. P., Bell A. S., Brown D. G., Dodd P. G., Hewson C., Hughes S. J., Phillips C., Ryckmans T., Smith R. T., van Hoorn W. P., Cohen P., Jones L. H. (2013). Chem. Biol. Drug Des..

